# Sputtering: A Versatile Technology to Deposit Multifunctional Protective Coatings

**DOI:** 10.3390/ma19163427

**Published:** 2026-08-12

**Authors:** Nuno Miguel Figueiredo, Bruno Martins, Eduardo Luís Silva, Albano Cavaleiro, Filipe Fernandes

**Affiliations:** 1 CEMMPRE—Centre for Mechanical Engineering, Materials and Processes, ARISE—Advanced Production and Intelligent Systems, Department of Mechanical Engineering, University of Coimbra, Rua Luís Reis Santos, 3030-788 Coimbra, Portugal; albano.cavaleiro@dem.uc.pt (A.C.); fid@isep.ipp.pt (F.F.); 2 LED&MAT-IPN—Laboratório de Ensaios, Desgaste e Materiais, Instituto Pedro Nunes, Rua Pedro Nunes, 3030-199 Coimbra, Portugal; 3 Durit Coatings, Parque Industrial de Taveiro 41 e 42, 3045-508 Taveiro, Portugal; innovation@duritcoatings.pt; 4 CIDEM, ISEP—Polytechnic of Porto, Rua Dr. António Bernardino de Almeida, 4249-015 Porto, Portugal

**Keywords:** PVD, sputtering, protective coatings, decorative, high-temperature lubrication, temperature sensing

## Abstract

Among the vast array of technologies available for surface modification of materials, sputtering emerges as one of the most versatile methods through coating deposition. Included in the family of physical vapor deposition (PVD) techniques, sputtering allows the production of coatings with a great variety of characteristics, based on a bottom-up approach that forms coatings from individual species (atoms or ions). This versatility is achieved by controlling: (i) the layer architecture, from monolithic to multilayers, (ii) the structures, from amorphous to nanocrystalline or nanocomposite, until highly crystallized, including epitaxial; (iii) the morphologies, from very porous through columnar or zig-zag to very dense and featureless; (iv) the chemical composition, allowing the deposition of metallic, polymeric, ceramic or composite materials types. In this paper, after a brief introduction of sputtering as a deposition technology, we will review the application of sputtering for depositing protective coatings to which an extra functionality is provided: (a) aesthetic color; (b) high-temperature lubrication; and (c) temperature sensing ability.

## 1. Introduction

Physical vapor deposition (PVD) encompasses a group of vacuum-based coating techniques widely used to deposit thin films with tailored properties on diverse substrates. Among the various PVD processes—such as sputtering, evaporation and cathodic arc deposition—sputtering stands out as one of the most versatile due to its ability to deposit a wide range of materials with precise control over composition, thickness, and microstructure [1,2]. This versatility enables its application across numerous industries, with coatings designed for decorative, tribological, thermal barrier, high-temperature, optoelectronic, semiconductor, solar, biomedical, anti-fingerprint, self-cleaning, sensor, catalytic, and smart surfaces. Each application area holds strong technological and commercial relevance. Sputtering is particularly well-suited for protecting applications that demand surfaces with superior mechanical [3], tribological [4], optical [5], and functional properties [6], due to its ability to produce smooth, defect-free films with excellent adhesion and uniformity over complex geometries [7,8,9].

Compared to traditional coating techniques such as electroplating and painting, sputtering offers multiple advantages for protective applications [7,10,11]. These include the elimination of hazardous chemicals, thereby minimizing environmental impact and improving workplace safety, as well as superior coating hardness, wear resistance, and corrosion/oxidation resistance, which translate into longer service lifetimes and improved performance in harsh conditions—including outdoor or high-humidity environments. Such attributes also contribute to color stability over time in decorative applications.

While chemical vapor deposition (CVD) can be used alongside sputtering to enhance functionality—particularly for conformal or complex materials—it typically requires high processing temperatures [12,13], which can induce thermal stress in both coatings and substrates. This limits its use on thermally sensitive materials. In contrast, sputtering can be performed at significantly lower temperatures [14], broadening its applicability to substrates such as polymers and electronic devices [15]. Another key advantage of sputtered coatings is their biocompatibility [16] and resistance to high-temperature cleaning and sterilization, making them particularly suitable for applications such as biomedical instruments and cutlery [17,18].

**Figure 1:**
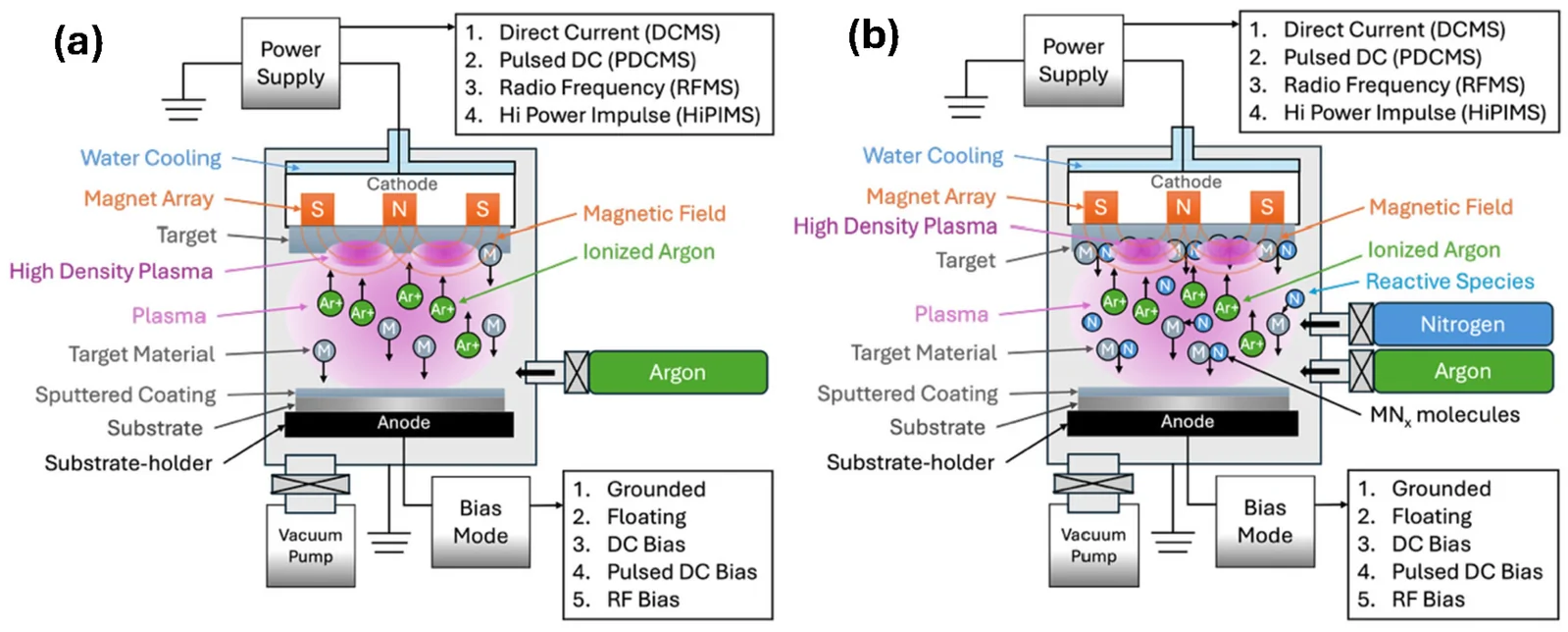
Schematic representation of the magnetron sputtering deposition method: (**a**) Conventional magnetron sputtering—in this process, a metallic or compound target is bombarded by ions from an inert gas (usually Ar). These energized ions eject atoms or particles from the target, which are then deposited onto the substrate surface, replicating the composition of the target material; (**b**) Reactive magnetron sputtering—in this method, a metallic target (M) is bombarded in a mixed atmosphere containing an inert gas (Ar) and a reactive gas (such as N_2_ or O_2_). The reactive gas chemically reacts with the target atoms, forming a compound film (MN_x_ or MO_x_) on the substrate surface. The film’s stoichiometry is controlled by adjusting the ratio between the inert and reactive gases within the deposition chamber.

Among sputtering methods, magnetron sputtering is especially valued for its high efficiency, uniformity, and flexibility in producing advanced coatings with nanoscale control. Figure 1 illustrates the operating principle of magnetron sputtering.

Magnetron sputtering provides atomic-level control over film growth, enabling the fabrication of advanced coating architectures—including nanostructured, nanocomposite, and multilayer systems—with finely tuned surface functionalities and minimal impact on substrate integrity. Nearly all elements in the periodic table, as well as complex alloys and compounds, can be deposited using this technique.

**Figure 2:**
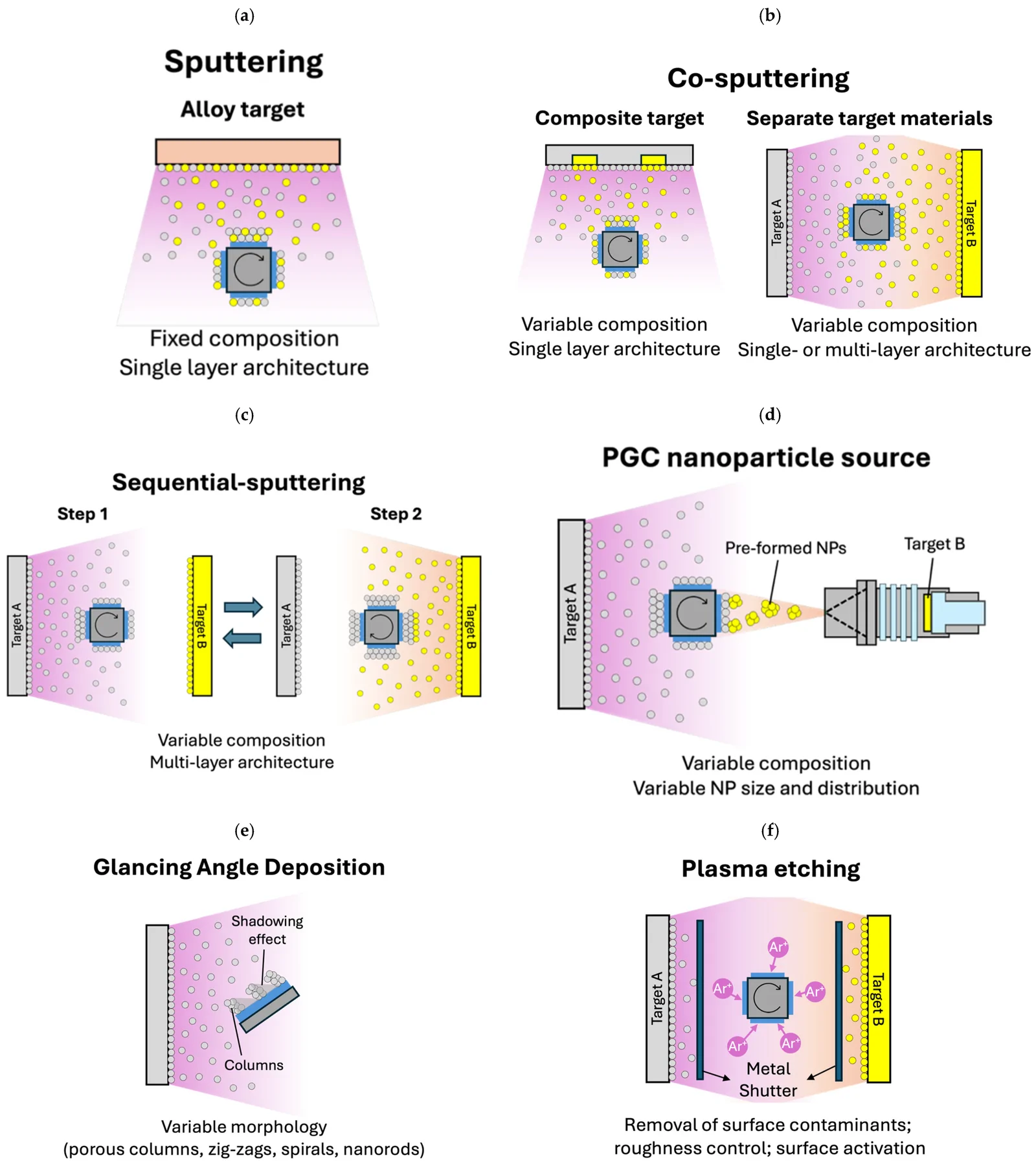
Schematic illustration of common sputtering-based strategies for depositing advanced coatings: (**a**) Sputtering from an alloy target; (**b**) Co-sputtering using either a composite target or separate targets; (**c**) Sequential sputtering by alternating between different targets; (**d**) Integration of a plasma gas condensation (PGC) nanoparticle source into either of the two previous strategies; (**e**) Glancing/Oblique angle deposition (GLAD); (**f**) Plasma etching treatment. Substrates are represented by blue rectangles positioned on the grey substrate holder.

Figure 2 provides a schematic overview of the main sputtering deposition routes. The deposition of doped and composite thin films can be readily accomplished by sputtering alloy targets (Figure 2a) or by co-sputtering from composite targets or multiple sources (Figure 2b) [19]. Additionally, ultrathin layers, multilayers, and complex nanostructures can be engineered using advanced techniques such as co-sputtering (Figure 2b) [20], co-sputtering followed by thermal annealing [21], sequential sputtering (Figure 2c) [4,22], plasma gas condensation (PGC) (Figure 2d) [23], glancing angle deposition (GLAD) (Figure 2e) [24], reactive gas pulsing [25], pulsed deposition [26], deposition of metal layers over templated substrates [27], and thermal embedding of metal nanostructures into amorphous matrixes [28]. A major advantage of magnetron sputtering is its ability to operate at low temperatures (<80 °C), making it especially suitable for coating temperature-sensitive substrates such as polymers and porous materials [5,26].

Table 1 compares the main sputtering deposition techniques in terms of their advantages, limitations, representative coating architectures, and typical applications.

At its simplest, sputtering allows for the deposition of single layers, often only a few nanometers thick, by exposing a substrate to a sputtered flux of a single target material. These monolithic coatings are widely used for protective, decorative, and functional purposes. By rotating a substrate in front of one or more targets, high uniformity and excellent thickness control can be achieved, especially when combined with substrate biasing [29]. Those coatings can be deposited at an oblique angle (Figure 2e) where the sputtered material arrives at the substrate from a non-perpendicular angle. This geometry induces the growth of columnar structures with unique anisotropic properties [30]. When combined with substrate rotation or oscillation, complex three-dimensional nanostructures such as helices or zigzag columns can be fabricated [31]. These coatings can have enhanced surface area [32], controlled porosity [33], and direction-dependent optical [34,35] or mechanical properties [36], making them useful for sensors, catalysts, and photonic devices [30,35].

Sputtering is widely used to deposit multilayer coatings, consisting of alternating nanometric layers of different materials. These layers can be engineered to create superlattices with enhanced mechanical [37] and optical properties [38] or specific magnetic behaviors [39]. In sputtering systems equipped with multiple cathodes, multilayers can be deposited by periodically alternating the active target during deposition (Figure 2c) or by rotating the substrate at controlled speeds in front of two or more targets (Figure 2b). This approach has been successfully applied to produce hard coatings [40].

Beyond single layers and multilayers, sputtering also allows for the formation of nanocomposite coatings, which are more complex architectures composed of a matrix material embedded with nanoscale inclusions of a secondary phase [21,41]. These nanostructures can exhibit unique combinations of properties that are not found in conventional materials, such as high hardness with toughness or electrical conductivity combined with transparency [42]. Several advanced sputtering strategies have been developed to fabricate such nanocomposites with tailored nanoscale features. One such approach is co-sputtering (Figure 2b), in which two or more targets made from different materials are sputtered simultaneously [29]. By adjusting the power applied to each target, the relative composition of the resulting film can be finely tuned. This method enables the deposition of solid solutions [4], metal–dielectric nanocomposites [21], and films with gradient or homogenous dispersions of multiple phases [43]. Co-sputtering is especially useful for designing nanocomposites with embedded nanoparticles or to create amorphous–crystalline hybrid structures that provide superior mechanical [44] or electrical performance [45]. Another approach is sequential sputtering (Figure 2c), where the deposition process alternates between different targets in a controlled sequence [43]. This technique is ideal for forming well-defined multilayer nanocomposites [46] or layer-by-layer assembled films where the interlayer interfaces play a critical role in determining the material’s behavior. It is particularly effective for creating diffusion barriers [47], heterostructures [48], coatings where different phases need to remain chemically distinct yet structurally integrated [49], or nanocomposites with embedded periodically distributed metal nanoparticles [22]. Reactive sputtering introduces a reactive gas (e.g., N_2_, O_2_, CH_4_, etc.) into the sputtering chamber allowing the deposition of semiconductor or insulating materials with a wide range of compositions (Figure 1b) [9,50]. By “pulsing” the gas flow instead of maintaining a constant reactive atmosphere, it is possible to achieve better control over film stoichiometry and interface sharpness and to reduce target poisoning. This technique is valuable for producing oxide–nitride composite films or for precisely tuning the chemical composition of nanocomposite coatings where the balance between metallic and non-metallic phases is critical [25,51]. Plasma gas condensation represents a different approach in which sputtered atoms nucleate in the gas phase to form nanoparticles before arriving at the substrate (Figure 2d) [23,41,52]. These nanoparticles can then be embedded into a matrix or deposited as a self-organized nanostructured layer. This bottom-up approach enables the creation of nanocomposites with a high density of nanoscale inclusions and a high degree of tunability over particle size and distribution.

Despite its widespread adoption and numerous advantages, continuous improvement of the sputtering process remains a topic of research and innovation. Efforts have focused on optimizing key parameters such as plasma ionization levels [53], deposition rate [54], energy efficiency [55], sputter flux modulation [56], film adhesion [57] and interfacial adhesion [58]. One persistent challenge in conventional sputtering systems is the presence of “dark zones”—regions inside the chamber where film deposition is insufficient or completely absent [12]. Minimizing or eliminating these zones increases coating uniformity and can be achieved through several strategies, including: (i) unbalanced magnetron sputtering, which can generate high ion currents capable of reaching dark zones when combined with an appropriate substrate bias voltage [59]; (ii) the use of high-power sources that produce increased ion fluxes, coupled with synchronized substrate biasing, thereby enhancing ion penetration and reducing atomic shadowing effects [60]; and (iii) the implementation of special rotating substrate holders, such as planetary systems with two- or three-fold rotational degrees of freedom, which improve angular flux distribution on components with moderate to complex geometries [61]. Another critical area of development is the improvement of target material utilization. Traditional sputtering configurations often suffer from low target usage efficiency, leading to material waste and increased production costs [13]. Advances such as the refinement of magnetic field configurations in magnetron sputtering systems [62] and the redesign of target geometries [63] have demonstrated the potential to significantly enhance both deposition rate and target material utilization.

In summary, sputtered coatings can be precisely tailored in terms of composition, structure, microstructure, morphology and architecture to meet the demanding requirements of next-generation technologies in areas ranging from aerospace [64] and medical implants [17] to energy devices [65] and optoelectronics [66]. Consequently, the rapid expansion of sputtering research has been accompanied by a growing number of review articles addressing diverse aspects of the field.

Over the past decade, review articles on sputtering have addressed its progress and recent advances [14,67,68], coating uniformity [69], process efficiency [70], process physics [53], process modeling [71,72], hydrophobic coatings [73], magnetic coatings [74], multilayer coatings [75], coatings on fabrics [76,77,78], coatings grown with metal-ion irradiation [70,79,80,81], electrocatalysts for water electrolysis [82,83,84], environmental remediation [14,85], metal-oxide-based displays [86], photovoltaics [87,88], electrochemical energy storage (including batteries and supercapacitors) [89,90,91,92,93,94], energy conversion technologies (including fuel cells and hydrogen-related applications) [14,89,95,96,97], self-lubricating coatings [98], thermal barrier coatings [99], membranes for gas and water treatment [100], gas sensors [101,102], anti-microbial coatings [103], biomedical devices [14,104], and high-entropy alloys (HEAs) [105,106,107].

Although recent reviews have addressed numerous aspects of sputtering technology, some application areas remain insufficiently covered. Therefore, this review focuses on three representative classes of multifunctional protective coatings requiring an updated and comprehensive assessment: (i) decorative coatings, where aesthetic performance is integrated with durability; (ii) low-friction coatings for high-temperature applications, where tribological performance is combined with thermal stability; and (iii) high-temperature sensing coatings, where protective coatings are engineered to provide in situ monitoring capabilities. These topics exemplify distinct strategies for extending the functionality of sputtered coatings beyond conventional protection, while highlighting recent advances in deposition strategies, coating architectures, and design approaches aimed at optimizing performance for specific applications.

## 2. Methodology

The publications included in this review were identified through a literature search conducted in the Scopus database using combinations of relevant keywords. The search strategy was organized into three thematic sections:

- Section A—Aesthetic as an Extra Functionality in Protective Coatings: “Decorative coating” AND “sputtering”; “Structural color” AND “coating” AND “sputtering”; “Plasmonic” AND “color” AND “sputtering”.
- Section B—The Extra Challenge of Low Friction at High Temperatures in Protective Coatings: “Low friction” AND “high temperature” AND “sputtering”.
- Section C—The Emergence of Thin-Film Sensors as a Solution for High-Temperature Measurement in Protective Coatings: “High Temperature measurement” AND “sputtering”; “High Temperature sensor” AND “sputtering”; “Temperature monitoring” AND “sputtering”; “Cutting Temperature” AND “sputtering”; “Machining Temperature” AND “sputtering”.

Only studies reporting coatings deposited by sputtering techniques were considered for inclusion in this review. Additionally, priority was given to publications published within the last decade to ensure that the review reflects the current state of the art, while a limited number of earlier studies were also included where relevant. To ensure the inclusion of scientifically established contributions, publications with no citations were excluded, as were publications lacking sufficient experimental information regarding coating design or deposition parameters.

## 3. Section A—Aesthetic as an Extra Functionality in Protective Coatings

Decorative PVD coatings achieve coloration through two primary mechanisms: intrinsic coloration and structural coloration. Intrinsic colors are characterized by uniformity and constancy, regardless of the coating thickness, whereas structural colors may vary significantly depending on coating design characteristics and/or viewing angle. Both types of coatings can be effectively deposited using the sputtering techniques illustrated in Figure 2.

### 3.1. Coatings with Intrinsic Colorations

Intrinsic colors originate from the fundamental electronic properties of the coating material, where the absorption and reflection of specific wavelengths of light determine the perceived color. The absorption processes depend on several mechanisms that arise from the interaction of light with both optical phonons and the outermost electrons. These mechanisms are classified into four main categories: *Reststrahlen* absorption, interband transitions, absorption due to impurities, and intraband transitions (also known as free-carrier absorption) [7,108,109]. Among these, free-carrier absorption is the primary mechanism responsible for the coloration of many protective decorative coatings such as nitrides and carbonitrides. These materials possess a completely filled valence band and an empty conduction band, separated by a band gap. Their electronic properties primarily depend on chemical composition (stoichiometry and impurity content) and crystalline structure. Variations in these parameters cause shifts in the optical band gap, altering the absorption edge and reflectivity of the films across the visible wavelength range, thereby producing different colors. The surface morphology and microstructure of the coatings, influenced by thickness and deposition conditions, can also affect their perceived color and brightness due to surface and volume scattering.

Industrial decorative coatings with intrinsic colors are commonly produced using materials such as titanium nitride (TiN), zirconium nitride (ZrN), titanium carbonitride (TiCN), zirconium carbonitride (ZrCN), titanium aluminum nitride (TiAlN), titanium aluminum chromium nitride (TiAlCrN), chromium (Cr), chromium nitride (CrN), and diamond-like carbon (DLC). Table 2 summarizes this information, highlighting the colors typically achieved in each case. A brief overview of their production strategies is provided below.

The properties of many functional coating materials deposited by vacuum techniques, particularly nitrides, are known to be adversely affected by the incorporation of contaminants such as oxygen [7,110]. To achieve high-quality nitride coatings, specific strategies must be employed. Some approaches, such as the use of expensive ultrahigh-vacuum (UHV) systems [111] or energetic ion bombardment from a separate source during deposition [112], are typically limited to laboratory-scale applications. Industrial-scale feasible strategies may include chamber baking at 250–450 °C [7,113] and prolonged or energetic plasma etching treatments [113,114] prior to deposition, as well as substrate heating above 200–300 °C [114,115,116] and maximizing target power [9,111,113] during deposition. For low-temperature operations, required for sensible substrates such as polymers, the most effective approaches often involve reducing the deposition pressure [111], utilizing pulsed DC [9] or RF [117] substrate bias during deposition, and, whenever possible, minimizing the target-to-substrate distance [111]. These strategies enhance the energy and/or flux of the sputtered metal nitride (MeN) particles, significantly improving the quality of the deposited nitride coatings. On the other hand, for polymeric substrates, to prevent coating cracking and delamination during the cool-down step, the substrate temperature should ideally remain below ~80 °C during deposition. This can usually be achieved by reducing the target power [5] or employing a pulsed deposition process [26,118].

Nitride coatings such as TiN, ZrN and CrN are commonly deposited via reactive magnetron sputtering (Figure 1b) in an atmosphere of Ar and N_2_. The physical and chemical properties of the nitride coatings can be controlled by adjusting the synthesis parameters, with one of the most critical factors being the N_2_/Ar flow ratio. This ratio influences the degree of target poisoning and the stoichiometry of the deposited coatings [9,11,108,116,119,120,121,122,123,124,125,126,127,128,129,130]. Golden TiN, yellow ZrN, and grey CrN coatings are typically achieved in stoichiometric MeN (when the Me/N ratio is close to ~0.9–1.1), which corresponds to N_2_ flow rates near the poisoning point. It should be noted that the minimum nitrogen partial pressure required for CrN phase growth is approximately ten times higher than that for TiN films, due to the difference in nitrogen adsorption rates between Ti and Cr. For N_2_ flow rates below the poisoning point, golden-to-brownish, yellow-to-grey and grey-to-silver colors are formed in the substoichiometric TiN_1−x_, ZrN_1−x_ and CrN_1−x_ coatings, respectively. For N_2_ flow rates above the poisoning point, where the composition of discharge gases changes rapidly and deposition rates progressively decrease, the formation of overstoichiometric MeN_1+x_ and/or the incorporation of oxygen contaminants in the coatings may be favored, resulting in bronze-colored TiN_1+x_, brass- or nickel-colored ZrN_1+x_, and dark-grey CrN_1+x_ coatings.

The deposition of TiCN and ZrCN coatings by reactive sputtering (Figure 1b) is commonly performed using a Me target (Ti or Zr) under an atmosphere of Ar, N_2_ and a hydrocarbon gas such as methane (CH_4_) [131,132], acetylene (C_2_H_2_) [133,134], or butane (C_4_H_10_) [135]. Alternatively, a single MeC target (Figure 2a,b) or dual Me + C targets (Figure 2b) can be used under an Ar + N_2_ atmosphere [136]. The most critical deposition parameter influencing the chemical composition, structure, and color of the coatings is the partial pressure of the reactive gas(es). In the first approach, a common strategy to achieve the desired colors in Table 2 is to maintain a fixed Ar + N_2_ flow rate while progressively increasing the hydrocarbon flow rate step by step for each deposition. In the second approach, using a fixed MeC target condition, increasing the N_2_ flow allows for variation in the N content of the coatings. Alternatively, when using two Me + C targets, both the C target power and N_2_ flow can be adjusted to achieve the desired tonalities.

The deposition of TiAlN and TiAlCrN coatings can be achieved by reactive sputtering using either individual metal targets (Ti and Al, or Ti, Al and Cr) [137,138,139] or composite targets (e.g., a Ti target with Al and/or Cr pellets evenly distributed across the racetrack) in an Ar + N_2_ atmosphere [4,140,141]. Typically, the Ar + N_2_ gas flow remains fixed, while the metal composition in the coatings is controlled by adjusting the power applied to each target, varying the number of pellets incorporated into the targets, or using a combination of both. Coatings with approximately 50 at.% N and varying Me element compositions are commonly achieved. TiAlCrN coatings generally exhibit colors ranging from dark grey to matte black, sometimes with subtle purple or bronze tints, depending on the specific composition and deposition conditions. The presence of Cr enhances oxidation resistance and tends to darken the coating compared to standard TiAlN.

A perfect chrome finish should have a lightness L* value as high as possible (typically 80–90) to maintain its reflective nature, while a* and b* values should remain close to zero to prevent unwanted color shifts (in the CIE L*a*b* color space, L* denotes lightness from 0 (black) to 100 (white), while a* and b* represent the green–red and blue–yellow color axes, respectively, with negative and positive values corresponding to green/red and blue/yellow, respectively). Chromium with a metallic silver appearance can be readily achieved through sputtering under an Ar atmosphere on various substrates at high deposition rates [10,11,57,126,142,143]. However, the exact color values may vary depending on factors such as microstructure, surface roughness, coating thickness, and the underlying substrate. Special attention is required for polymeric substrates, where plasma etching pre-treatment (Figure 2f) is essential to enhance coating adhesion and minimize defects such as buckling, cracking, and pinholes.

A perfect black color is characterized by a lightness L* as low as possible while preserving a metallic gloss and a* and b* values close to zero a. High-quality black decorative DLC coatings can be effectively achieved through sputtering, using a single C target under either a pure Ar atmosphere (Figure 1a) [144,145] or under a mixed Ar + hydrocarbon atmosphere (Figure 1b) [146]. The first approach results in pure amorphous coatings with lower deposition rates, while the second yields hydrogenated amorphous carbon coatings with higher deposition rates and better control over the H content. DLC is inherently amorphous, consisting of both sp^2^ (graphite-like) and sp^3^ (diamond-like) bonded carbon. Its unique properties are primarily determined by the sp^2^/sp^3^ ratio and hydrogen content. In magnetron-sputtered DLC coatings, small amounts of hydrogen help reduce friction, but minimizing hydrogen content enhances the diamond-like characteristics. Pure DLC coatings (low H content, high sp^3^) typically exhibit a deep black color with a glass-like appearance. In contrast, H:DLC coatings (higher H content, more sp^2^) tend to appear black with slight brownish or greyish undertones, depending on thickness and deposition conditions. DLC coatings often exhibit high internal stresses, which can compromise adhesion to the substrate. To mitigate this, an adhesion-promoting interlayer, such as Cr, Ti or Zr, is commonly applied. On the other hand, using a carbon-containing gas like methane or acetylene can lead to contamination of the deposition chamber, requiring frequent cleaning to maintain proper pumping efficiency and ensure good adhesion of the layers to the substrate.

### 3.2. Coatings with Structural Colorations

Structural colors arise from the interaction of light with nanostructures within the coating. These colors are produced through optical phenomena such as interference, diffraction, absorption, and scattering, rather than being inherent to the material’s composition.

#### 3.2.1. Colors Due to Interference Effects

**Figure 3:**
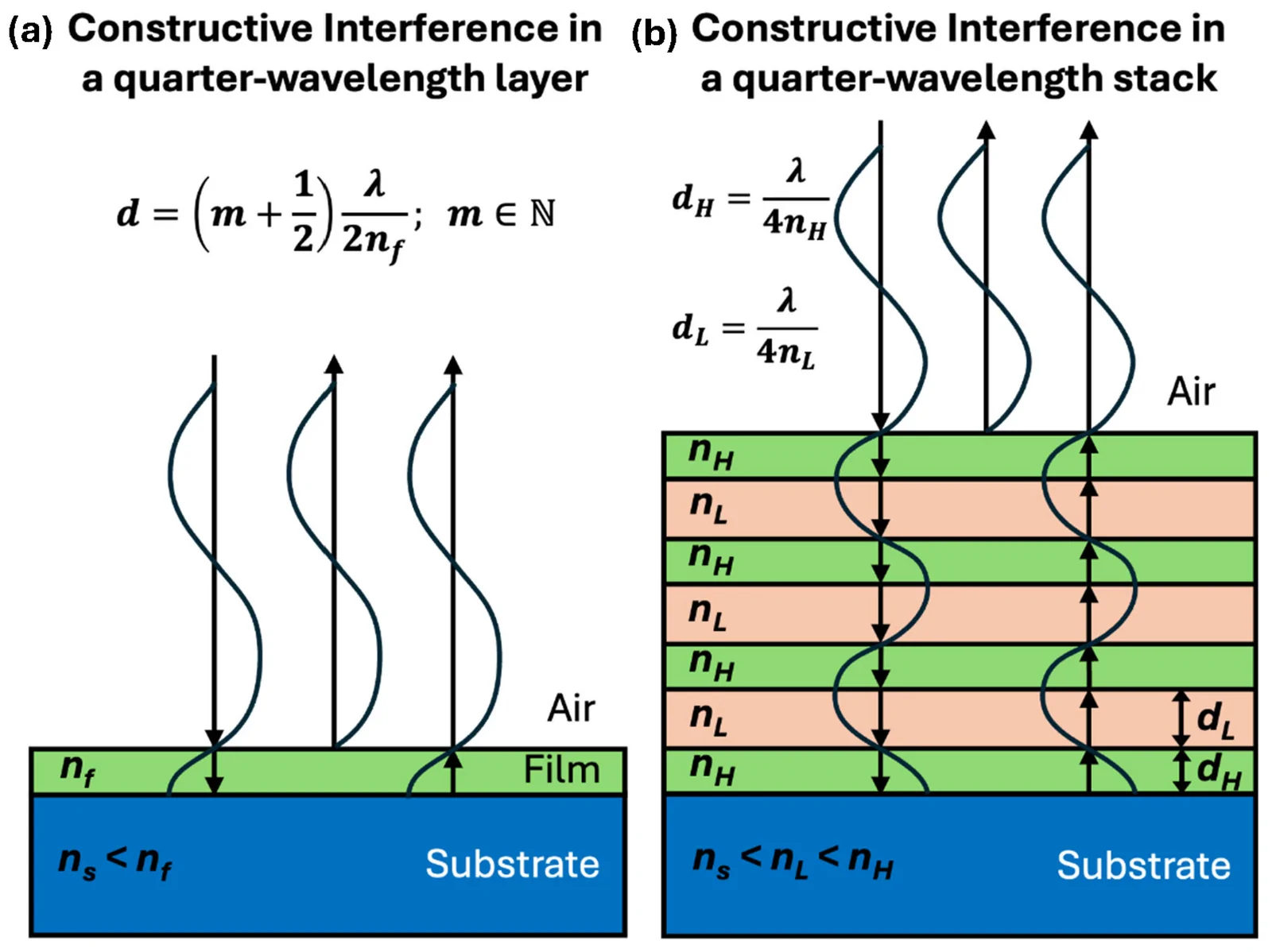
Schematic illustration of the interference effect in semitransparent films on a reflective substrate, demonstrating different design strategies: (**a**) a monolayer film; (**b**) a multilayer structure composed of alternating high- and low-refractive-index materials. Black arrows indicate the propagation direction of the incident and reflected electromagnetic waves at the different interfaces.

The most common method for achieving structural coloration in PVD coatings is through thin-film interference, where transparent or semitransparent oxide layers selectively enhance certain wavelengths of reflected light. The thickness of the film follows the quarter-wavelength condition (d=λ/4n), where λ is the targeted interference wavelength and *n* is the refractive index of the layer. This principle is illustrated in Figure 3a, where the single-layer film is designed to induce constructive interference at the targeted wavelength, enhancing reflectance in the desired spectral region.

Decorative coatings exhibiting a full spectrum of interference-based colors can be produced by (reactive) magnetron sputtering (Figure 1b) using thin layers of transparent or semitransparent materials such as titanium dioxide (TiO_2_) [147], titanium oxynitride (TiON) [118], AlN [148], niobium oxide (Nb_2_O_5_) [149], and tantalum oxide (Ta_2_O_5_) [150]. However, single-layer interference coatings generally exhibit limited color saturation because their reflectance maxima are relatively low and spectrally broad. Consequently, more advanced optical architectures have been developed to enhance color purity, increase reflectance, and expand the achievable color gamut.

A more advanced approach to structural coloration involves the use of distributed Bragg reflectors (DBRs), also known as dielectric mirrors or quarter-wavelength stacks, where alternating layers of high- and low-refractive-index materials create vibrant, tuneable colors. In Figure 3b, the design of a multilayer Bragg reflector is demonstrated. Typically, the stack consists of an odd number of layers, with the first and last layers selected to have a high refractive index. Each interfacial region causes partial reflection of an optical wave. When the wavelength is close to four times the optical thickness of the layers, the various reflected waves interfere constructively, making the layers function as a high-quality reflector. The performance of these coatings, including reflectivity and bandwidth, is influenced by the number of alternating layers and the refractive index contrast between the materials. Generally, a larger refractive index contrast results in higher reflectivity and a wider bandwidth, whereas an increased number of layer pairs enhances the reflectivity while narrowing the bandwidth. Despite this method traditionally being used for creating optical filters or broadband optical reflection across the Vis-NIR range [151,152,153], it enables the creation of bright colors across the visible spectrum, with applications in high-end decorative surfaces and optical coatings [153,154]. The deposition of alternating material pairs can be readily achieved through sequential (reactive) magnetron sputtering using two target materials (Figure 2c). Alternatively, the deposition of a single material with alternating dense/porous microstructures can be achieved by adjusting the deposition angle (normal or oblique) from a single target material using (reactive) magnetron sputtering (Figure 2e). In the oblique angle deposition, at higher incident vapor flux angles, nanoporous films with more inclined columnar structures can form due to the self-shadowing effect and limited mobility of incoming atoms. These films typically exhibit a lower refractive index compared to the dense films of the same materials. Very few studies in the literature focus on decorative coatings based on Bragg reflectors; however, some notable examples of high- and low-refractive-index material combinations for optical applications include dense/nanoporous TiO_2_ [153], dense/nanoporous amorphous silicon (Si) [155], dense/nanoporous amorphous indium tin oxide (ITO) [154], TiO_2_/SiO_2_ [151,156], and Sb_2_S_3_/SiO_2_ [157]. Recent advances of this design include the use of aperiodic distributed Bragg reflector (A-DBR) structures [157], which achieve high-purity color bands that are independent of incident angle and polarization by suppressing off-band reflections.

**Figure 4:**
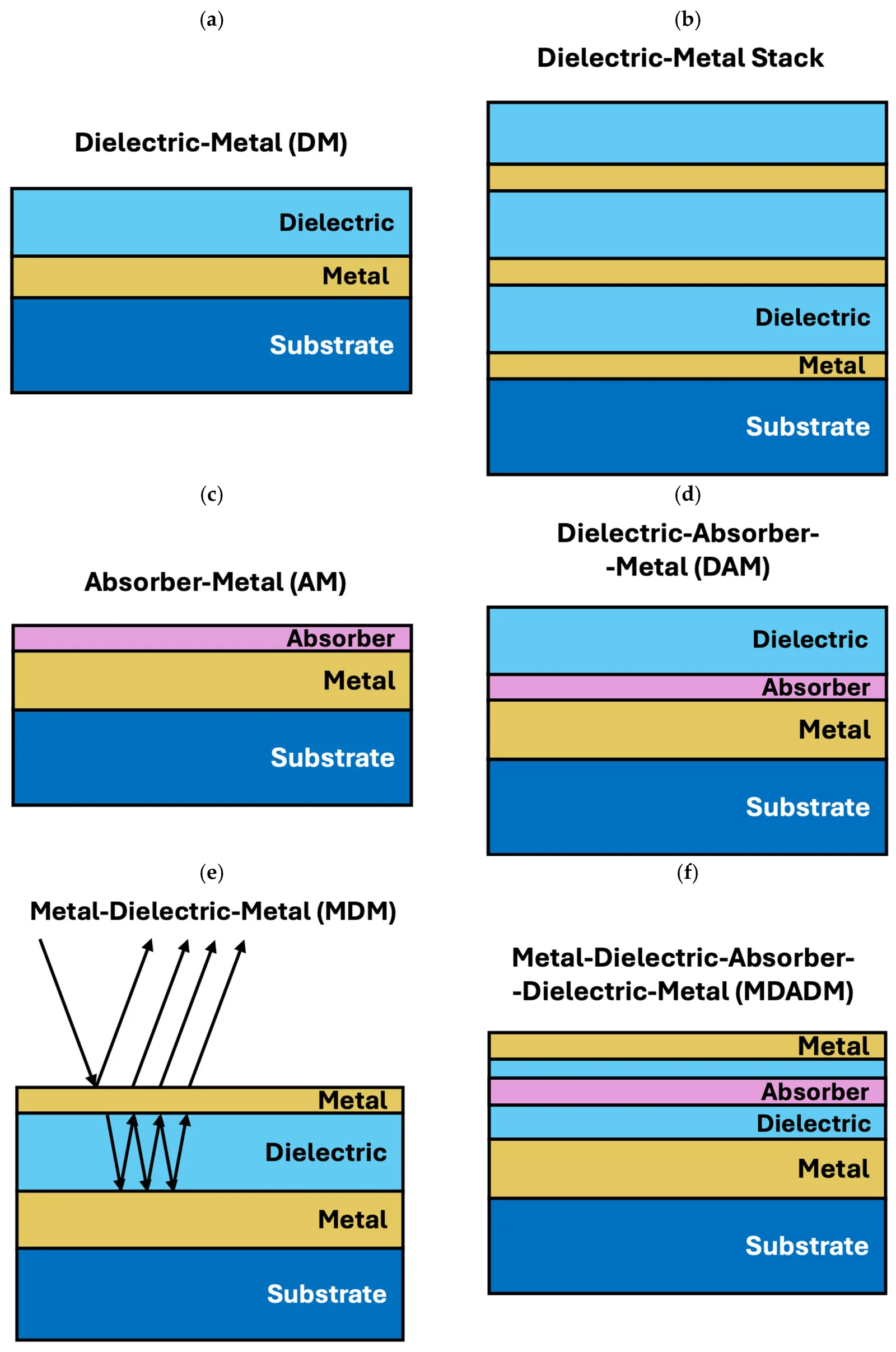
Schematic illustration of different design strategies for structural color coatings: (**a**) dielectric–metal; (**b**) dielectric–metal multilayer stack; (**c**) absorber–metal (AM); (**d**) dielectric–absorber–metal (DAM); (**e**) metal–dielectric–metal (MDM), also known as metal–insulator–metal (MIM), Fabry–Pérot resonator; and (**f**) metal–dielectric–absorber–dielectric–metal (MDADM).

Another approach to creating structural colors involves dielectric–metal reflectors, which consist of either a single dielectric–metal pair (Figure 4a) or multilayer stacks comprising alternating thin dielectric and ultrathin metallic layers (Figure 4b). The metallic component is typically selected based on its complex refractive index, which determines its reflectivity and absorption characteristics. A wide range of metals can be employed in these architectures, including excellent plasmonic metals such as gold (Au), silver (Ag), copper (Cu), and aluminum (Al); metals with moderate plasmonic performance, such as nickel (Ni), palladium (Pd), platinum (Pt), rhodium (Rh), ruthenium (Ru), indium (In), and gallium (Ga); and metals with relatively weak plasmonic responses, including chromium (Cr), titanium (Ti), zirconium (Zr), tungsten (W), and tantalum (Ta).

In single dielectric–metal structures (Figure 4a), the metallic reflector is typically thicker than 50 nm to ensure high reflectivity while minimizing the optical influence of the underlying substrate. In contrast, in dielectric–metal multilayer stacks (Figure 4b), the metallic layers are generally kept below 50 nm to remain significantly transparent, allowing light to penetrate the metal and interact with the underlying dielectric layers. This occurs because light can propagate a small distance inside a metal before being predominantly reflected. This wavelength-dependent characteristic length, known as the skin depth, is on the order of ~10–35 nm for most metals [158]. By tailoring the thicknesses of the metallic and dielectric layers, and, when required, the number of repeated metal–dielectric units, optical coatings with intense structural colors can be produced through constructive and destructive interference. Vibrant colors are typically achieved by tuning the first-order constructive interference maximum across the visible spectrum [159]. However, specific colors can also be obtained with thinner dielectric layers by positioning the first-order destructive interference minimum within the visible spectrum, even when the corresponding constructive interference maximum lies outside the visible range [159,160]. Unlike dielectric–dielectric interfaces, reflection at a dielectric–metal interface is associated with a wavelength-dependent phase shift arising from the complex refractive index of the metal. Consequently, the resonance condition depends on both the optical thickness of the dielectric layer and the reflection phase at the metal interface, making quarter-wave or half-wave conditions only approximate design guidelines.

High-quality, industrial-ready decorative coatings based on dielectric–metal multilayers can be achieved through sequential deposition using (reactive) sputtering, either with two target materials (Figure 2c) or a single target material combined with the reactive gas pulsing method (intermittent operation between the cases illustrated in Figure 1a,b). In this method, the reactive gas (usually O_2_ or N_2_) is pulsed into the deposition chamber in an on–off manner, enabling the sequential deposition of metal (pulse off) and oxide/nitride (pulse on). Examples of dual-target (reactive) sputtering depositions include the SiO_2_-Cu [161], Al_2_O_3_-Ag [162], WO_3_-Ag [163], ZnO-Ag [164], TiO_2_-Ag [165], AlN-Al [166], TiO_2_-Al/Ti [167], SnO_2_-stainless steel (sst) [168], and SiAlN_x_-sst [168] systems. Meanwhile, examples of single-target (reactive) sputtering using the pulsed reactive gas method include the TiO_2_-Ti [147,160,169], CrO_x_-Cr [159], AlN-Al [166], Ta_2_O_5_-Ta [170], Ag_2_O-Ag [171], and WO_x_-W [25,172] systems.

Recently, a class of optical coatings known as thin-film absorbers has attracted increasing interest for structural coloring. These coatings consist of an ultrathin high-loss dielectric film, typically only a few to a few tens of nanometers thick, deposited onto a highly reflective metallic layer (Figure 4c). The strong optical attenuation within the absorbing dielectric, together with the associated non-trivial reflection phase shifts, gives rise to pronounced resonant behavior in films that are much thinner than the wavelength of light. In these ultrathin structures, the phase accumulation associated with light propagation through the film is limited, while the reflection-induced phase change dominates the optical response. Consequently, these coatings exhibit reduced sensitivity to the angle of incidence. This architecture offers two major advantages over conventional interference-based coatings: reduced deposition time and lower color dependence on the viewing angle. Representative examples include TiAlN-sst [173], Si-Al [174], and Si-sst [175] fabricated by dual-target (reactive) sputtering and the MgZnCaO-MgZnCa system [176] produced by single-target reactive sputtering using the pulsed reactive gas method.

A further modification of this architecture consists of introducing a top low-loss dielectric layer above the lossy dielectric layer (Figure 4d), providing additional design flexibility to tune the optical response or enhance the environmental stability and wear resistance of the coating. When used as a protective layer, either very thin (<50 nm) or relatively thick (>1000 nm) dielectric layers are typically employed to minimize additional interference effects that could alter the color response during wear. Representative examples include the AlN-Si-Al system [174], fabricated by dual-target (reactive) sputtering, and the SiO_2_-Si-sst system [175], produced by single-target reactive sputtering using the pulsed reactive gas method. Interestingly, hard ceramic materials traditionally employed as intrinsic-color coatings, such as TiN, TiAlN, and AlN, can simultaneously fulfil the roles of reflective, absorbing, and dielectric layers, respectively, enabling the fabrication of highly wear-resistant optical coatings with vivid, angle-independent structural colors [177].

Another promising metal–dielectric architecture for structural color generation is the metal–dielectric–metal (MDM) structure, commonly known as a metal–insulator–metal (MIM) configuration and often described as a Fabry–Pérot (FP) cavity when the optical response is governed by thin-film interference (Figure 4e). In this coating design, the top metallic film is typically thin enough to allow partial transmission and reflection, while the bottom metallic film is usually highly reflective, acting as a mirror. This structure acts as an optical resonator, where multiple reflections within the dielectric layer produce interference effects that define its reflection and transmission properties. Constructive interference occurs when the wavelength is close to two times the optical thickness of the dielectric layer (where the optical thickness is the product of the refractive index by the thickness of the layer), generating resonant structural colors [178]. For wavelengths where the electric field is low at the semiabsorbing thin metal layer, high overall reflectance is obtained, corresponding to the reflectance peaks. Conversely, for wavelengths where the electric field is high at this semiabsorbing metal layer, low overall reflectance is observed, corresponding to the reflectance minima. Thus, for fixed thicknesses of the top and bottom metal layers—usually around 10 nm and 100 nm, respectively—the thickness of the dielectric layer determines the position of the reflection peak. Many optical coatings rely on Fabry–Pérot-type interference to achieve functionalities such as anti-reflection, high reflection, dichroism, and tuneable colors.

For decorative applications, conventional Fabry–Pérot cavities employing highly reflective metals, such as Ag, often exhibit limited color saturation and brightness due to insufficient broadband absorption. This limitation can be addressed by replacing the top reflective layer with a broadband absorbing metal, such as Ni, thereby enhancing the spectral selectivity and color purity of the reflected light [179]. Large-area fabrication of these multilayer structures can be achieved by sequential (reactive) magnetron sputtering (Figure 2c). Representative examples include Cr-SiO_2_-Cr [179], Au-SiO_2_-Al [180], Ag-SiO_2_-Ag [181,182], Ni-SiO_2_-Al [183], and Al-Al_2_O_3_-Al [184].

A recent development of the traditional MIM architecture is the implementation of multi-Fabry–Pérot cavities [179], which enables the generation of high-purity colors at the expense of increased viewing-angle dependence, a feature that can be advantageously exploited for the anti-counterfeiting of high-value industrial products.

Another promising modification of the conventional MIM design is the incorporation of an ultrathin absorbing layer within the dielectric cavity (Figure 4f). By positioning this layer at a region of high optical field intensity, an additional absorption band is introduced alongside the Fabry–Pérot cavity resonance. Combined with refractive-index engineering of the metallic layers, this approach enables the generation of highly pure red, green, and blue colors with bandwidths of approximately 100 nm and reflection efficiencies approaching 90% [185].

#### 3.2.2. Colors Due to Absorption and/or Scattering of Light

**Figure 5:**
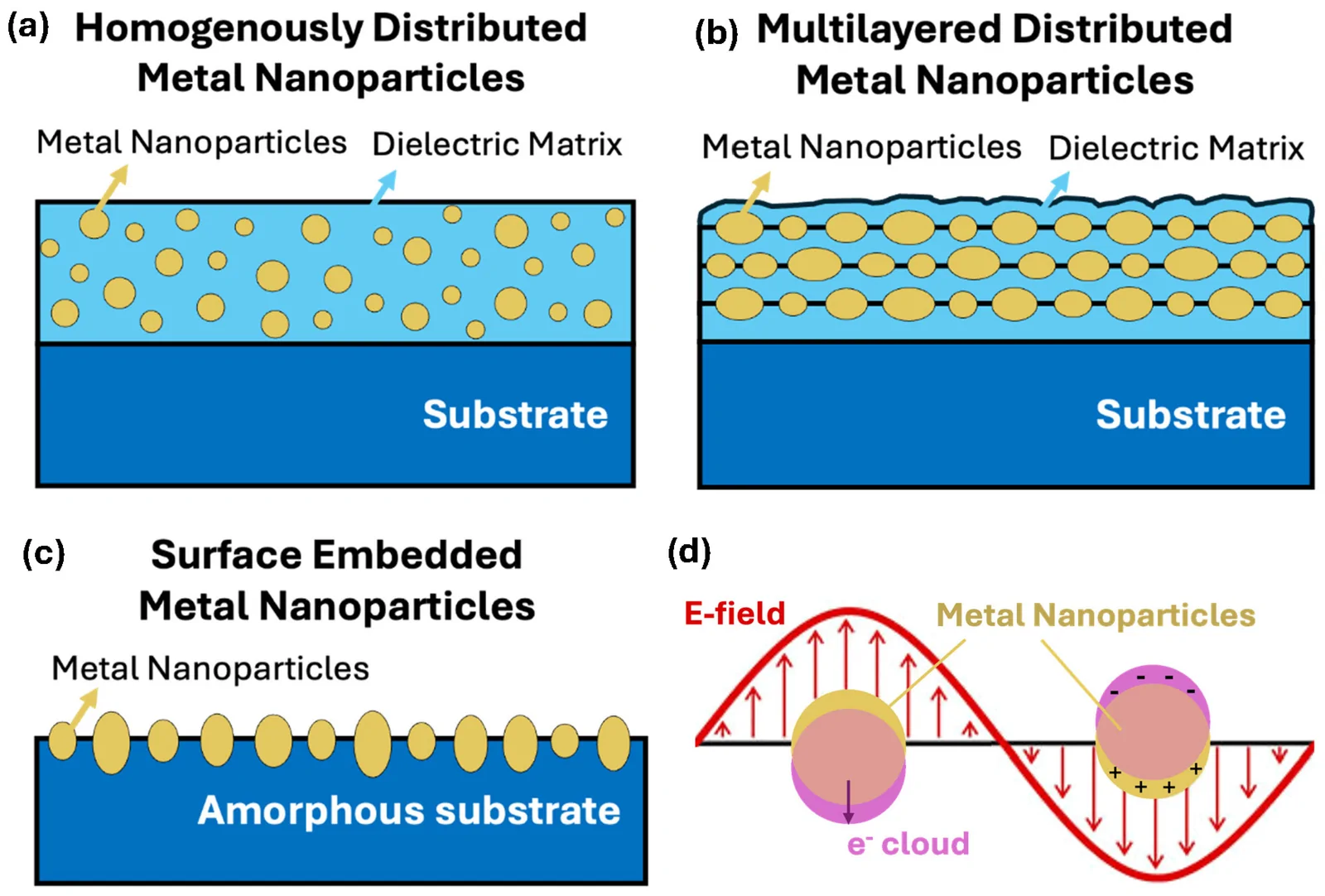
Schematic illustration of different design strategies for metal–dielectric nanostructured coatings: (**a**) monolayer of homogeneously dispersed metal nanoparticles within a dielectric matrix; (**b**) multilayer structure of metal nanoparticles embedded in a dielectric matrix; and (**c**) thermally embedded metal nanoparticles on the surface of an amorphous dielectric material. (**d**) Schematic illustration of the oscillation of a metal nanoparticle’s electron cloud upon excitation by incident light with a frequency close to the surface plasmon resonance frequency of the metal nanoparticle, with the red arrows indicating the local electric-field direction and amplitude.

Another promising strategy in the field of optics, including for decorative applications, is the development of nanostructured composites containing metal nanoparticles. The primary goal in this area is to promote the formation and distribution of nanoparticles with specific sizes, shapes, and arrangements, either at the surface, embedded within a host matrix, or as a combination of both [186]. Figure 5a–c illustrate such design alternatives.

The metals are capable of supporting surface plasmons (SPs), which are collective oscillations of free electrons in confined metal systems, when excited by external electromagnetic waves. For materials with dimensions well below the subwavelength scale, particularly nanoparticles, the plasmon oscillates locally around the particle, a phenomenon known as localized surface plasmon resonance (LSPR). The resonance condition occurs when the frequency of the incident light matches the frequency of the conduction electrons oscillating under the influence of the electromagnetic field, opposing a restoring force towards the equilibrium distribution (Figure 5d). At this resonance frequency, the electric field near the nanoparticle surface is significantly enhanced, and the optical extinction is substantially increased due to the large polarization associated with the collective electron oscillation. The decay of the plasmonic excitation can occur either through the radiative emission of a photon (light scattering) or non-radiatively (light absorption). Scattering dominates over absorption for nanoparticle sizes above approximately 50 nm. For sizes below this threshold, absorption becomes the dominant mechanism, with scattering gradually diminishing and becoming negligible for nanoparticles smaller than ~30 nm. The position and shape of the LSPR bands, which influence the intensity and range of achievable colors, depend on the plasmonic material, the size, shape, and distribution of the nanoparticles within the host matrix, as well as the dielectric properties of the matrix [158,187]. Concerning the plasmonic material, Au, Ag, and Cu each exhibit SP resonances in the visible spectrum at different frequencies, thereby affecting the positioning of the LSPR bands. Aluminum, on the other hand, exhibits a plasmon resonance in the visible spectrum only for nanoparticle sizes above ~50 nm—when higher-order multipoles are excited and the contribution of light scattering to the extinction becomes non-negligible—or in the case of non-spherical shapes. Regarding the influence of nanoparticle shape, and considering the simpler cases of spheroidal nanoparticles (which include both oblate/nanodisks and prolate/nanorods), a decrease in aspect ratio (defined here as the ratio between the short and long axis lengths) leads to a splitting of the plasmon resonance into a strongly red-shifted long-axis mode and a slightly blue-shifted short-axis mode. In terms of the host medium, the LSPR peak red-shifts with increasing refractive index, due to the buildup of polarization charges that weaken the overall restoring force on the dielectric side of the interface. Additionally, at higher volume fractions of metal nanoparticles, when the interparticle separation becomes smaller than the nanoparticle diameter, electromagnetic coupling between neighboring particles occurs, resulting in a progressive red-shift of the LSPR signal.

Plasmonic nanomaterials have been prepared by several sputtering techniques, including co-sputtering (Figure 2b) followed by thermal annealing [20], sequential sputtering (Figure 2c) [22], deposition of pre-formed metal nanoparticles by plasma gas condensation (also known as gas aggregation clusters source, or cluster gun, or nanoparticle gun—Figure 2d) [23], deposition of metal layers over templated substrates [27], oblique angle deposition (Figure 2e) [24], or thermal embedding of deposited metal nanostructures on the surface of amorphous materials [28]. Some examples of these materials and their colors are as follows: Au-Al_2_O_3_ (yellow, brown, pink, reddish, grey) [20,22,23,188], Au-TiO_2_ (red, brown, grey) [22,23,24,189,190], Au-AlN (reddish, brown) [191], Au-WO_3_ (yellow, brown, light blue, dark blue, purple, violet, grey) [21,192], Au-glass (reddish, pink) [28], Ag-SiO_2_ (yellow, orange, purple, blue) [193], Ag-fluorocarbon (yellow, orange, red-orange, purple, violet) [194], Au-, Ag- and Al-polystyrene (red, green, blue, orange, yellow, pink, purple) [27], Al-SiN and Al-aSi [195].

Drawbacks associated with these strategies include a reduction in hardness with increasing nanoparticle content, as well as coating degradation after exposure to high-temperature thermal treatments. In addition, among the plasmonic metals typically used, gold can be considered expensive for certain applications, while silver is often more difficult to stabilize due to its higher reactivity. To address these issues, alternative plasmonic refractory materials such as TiN, ZrN and HfN [196,197] have been proposed, where the nanoparticles are pre-formed by reactive magnetron sputtering under elevated pressures using a plasma gas condensation technique (Figure 2d). These materials show excellent thermal stability at elevated temperatures and are cheaper alternatives to the use of noble metals [198].

Recent developments have also focused on decorative coatings based on plasmonic nanostructures with increasingly complex geometries and arrangements, fabricated using either templated or template-free approaches. Representative examples of templated architectures include HfN nanodisks and nanocavities [199], as well as Al nanocavities and nanoumbrellas [200]. In contrast, notable template-free strategies include tuneable Ag nanowire arrays embedded in a SiO_2_ matrix, capable of generating the full visible color gamut [201,202]. These nanostructures were fabricated by one-step co-sputtering assisted by radio-frequency (RF) substrate bias, demonstrating the potential of scalable sputtering processes for producing complex plasmonic architectures.

#### 3.2.3. Hybrid Photonic–Plasmonic Approaches

Hybrid photonic–plasmonic architectures represent an emerging strategy for structural color generation, combining interference effects with localized or propagating plasmonic resonances. By integrating photonic cavities with plasmonic nanostructures, these architectures provide additional degrees of freedom for tailoring the spectral response, enabling improved color saturation, enhanced reflectivity, and, in some cases, reduced angle dependence compared with conventional interference-based coatings.

One approach consists of integrating plasmonic metallic nanoparticles on top of MIM resonators, allowing the simultaneous excitation of Fabry–Pérot cavity modes and localized gap plasmons. The coupling between these resonances enables vivid structural colors with reduced dependence on both the viewing angle and the polarization of the incident light [203].

Another strategy employs periodically perforated ultrathin metallic films containing arrays of nanoholes. In these architectures, the periodic nanostructure supports surface plasmon resonances that can be spectrally tuned across the visible range, producing narrow absorption bands and near-perfect optical absorption at selected wavelengths [184].

Hybrid photonic–plasmonic effects can also be achieved by depositing plasmonic metals onto porous dielectric templates. Representative examples include sputtered Au [204,205] and Cr [205] films deposited on porous anodic alumina, where the resulting color can be tailored by adjusting the pore height.

An alternative mechanism is based on polarizonic interference, which arises from the interaction between the localized resonant response of plasmonic nanoparticles and the broadband reflection of an underlying metallic or semimetallic film. Under appropriate conditions, destructive interference between these optical contributions produces a pronounced reduction in reflectivity at the plasmon resonance wavelength. Representative examples of co-sputtered nanocomposites include Au-SiO_2_ and Au-SiO_2_-TiO_2_ deposited on Al substrates [206], and Au-Al_2_O_3_ deposited on sst substrates [207], where the resulting color originates from the combination of plasmonic and interference effects. A representative example of a sequentially sputter-deposited nanocomposite is the Ag-ITO system deposited on Ti6Al4V substrates [208].

### 3.3. Comparative Assessment of Decorative Coatings Strategies

Table 3 compares the main decorative coating strategies, highlighting their representative architectures, color-generation mechanisms, optical characteristics, durability, advantages, limitations, and typical sputtering deposition approaches.

Among these strategies, intrinsic-color coatings remain the most industrially mature owing to their excellent durability, scalability, and compatibility with conventional sputtering processes. However, their relatively limited color palette restricts applications requiring highly saturated or unconventional colors. Structural-color approaches, including distributed Bragg reflectors (DBRs), metal–insulator–metal (MIM) resonators, and plasmonic architectures, overcome these limitations by enabling a much broader color gamut and higher color purity. Nevertheless, these approaches generally require more complex coating architectures, tighter process control, and, in many cases, exhibit increased sensitivity to the viewing angle.

Among the various structural-color strategies, thin-film absorber architectures are particularly attractive because they combine simple multilayer designs with excellent optical performance. Their use of ultrathin functional layers results in shorter deposition times, reduced material consumption, and lower manufacturing costs, while maintaining low color dependence on the viewing angle. Furthermore, the incorporation of a top dielectric layer can improve the chemical, mechanical, and optical stability of the coating throughout its service life. Since this architecture can be entirely based on hard ceramic materials, it also offers excellent wear resistance and is readily compatible with well-established industrial sputtering processes.

Hybrid photonic–plasmonic architectures represent another promising direction, combining interference and plasmonic effects to achieve a full visible color gamut and high color fidelity while offering the possibility of tailoring the viewing-angle dependence. However, these approaches remain at a relatively early stage of development, with limited published studies and greater fabrication complexity, requiring further research before widespread industrial implementation.

## 4. Section B—The Extra Challenge of Low Friction at High Temperatures in Protective Coatings

The growing demand for materials capable of withstanding extreme thermal environments, ranging from aerospace propulsion systems to industrial machining and energy generation, has driven extensive research into advanced lubrication strategies [209]. Unlike conventional liquid lubricants, which degrade at high temperatures, solid lubricants materials provide long-lasting, reliable performance under severe high temperature operating conditions [210]. The concept of self-lubrication revolves around the ability of materials to form a lubricating layer in situ, reducing/avoiding the direct contact between the sliding surfaces and thereby minimizing friction and wear [211]. The development of hard coatings with high-temperature adaptive behavior is crucial for applications requiring extreme thermal stability, wear resistance, and low friction over a broad temperature range [212]. The self-lubricating effect at high temperature can be achieved through various mechanisms [213]: (i) structural transitions for adaptive behavior; (ii) adaptive mechanisms using metal diffusion; and (iii) tribo-oxidation mechanisms. Each of these mechanisms plays a crucial role in determining the effectiveness of the coating under different environmental conditions. The former materials provide excellent lubrication in dry and in some cases humid conditions by allowing sliding between their weakly bonded atomic planes [214]. However, their effectiveness diminishes in oxidative environments above 500 °C, where they tend to degrade into non-lubricious oxides or simply evaporating. Nevertheless, transition-metal dichalcogenides encapsulated within oxides can help to maintain oxidation resistance while providing lubrication. In the second case, metal-diffusion-based adaptive coatings leverage the controlled migration of soft metals to the contact surface [215]. Diffusion is controlled by using barriers to regulate the rate at which the lubricant reaches the wear track, thereby extending the coating’s lifetime. In the third case, oxidation of specific elements within the coating results in the formation of lubricious oxides exhibiting a layered structure, reducing shear strength and providing effective lubrication at elevated temperatures [216]. The same types of barrier layers as for metal-diffusion-based adaptative coatings are being used to block the fast diffusion of the lubricious forming elements. In this subsection the strategies which should be applied to produce/customize self-lubricant coatings to work efficiently at high temperature are summarized.

### 4.1. Design of Coatings with Adaptive Mechanisms Based on Structural Transitions

In the pursuit of advanced low-friction surface engineering solutions, the development of coatings with adaptive behavior, those that respond to external stimuli through structural transitions, has become a leading research direction [217]. Unlike conventional coatings, which maintain fixed properties regardless of their environment, adaptive coatings dynamically alter their structure or composition to enhance performance under changing conditions. These changes are often triggered by mechanical stress, temperature, electric or magnetic fields, or chemical exposure. The key to designing such coatings lies in manipulating their intrinsic structure at the atomic or nanoscale level, enabling reversible or irreversible transformations that confer new or improved functionalities in service [218].

One well-documented phenomenon is the reorientation of initially random polycrystalline hexagonal lubricant solids, such as transition metal dichalcogenide (TMD) films (e.g., MoS_2_, WS_2_, WSe_2_, etc.) [219]. The alignment of the hexagonal basal planes parallel to the surface significantly reduces friction and wear due to their low shear resistance due to weak van der Waals interactions between basal planes. Those materials behave as excellent solid lubricants, with friction values reported in the range of 0.1 or even 0.01 in vacuum conditions [219,220]. Despite their unique tribological properties and successful application in renowned space missions such as the JWST, Hubble, CMD, and Galileo telescopes, the large-scale industrial use of pure TMD coatings remains limited. This is primarily due to: (i) their high porosity, which makes them vulnerable to moisture-induced degradation at room temperature and oxidation at high temperatures; and (ii) their low hardness, which limits their ability to withstand high mechanical loads during service [221].

Alloying TMDs with various metals or non-metals enhances their performance at increasing temperatures. However, their thermal resistance (<300 °C) and mechanical strength are still lower than what is required for high-temperature applications [222]. These coatings can be deposited through various approaches, including reactive sputtering (Figure 1b), co-sputtering from a compound target (Figure 2b), co-sputtering from a metal (e.g., Mo, W) and a chalcogen (e.g., S, Se) target (Figure 2b), or by sequential deposition of two metals (Figure 2c) followed by post-deposition sulfurization. In all cases, adhesion and gradient interlayers are commonly employed to promote strong bonding between the coating and the substrate. These self-lubricating mechanisms extend to amorphous carbon solids, where hexagonal structure crystallization occurs within tribological contacts. A notable example is the formation of a graphitic tribolayer in initially amorphous DLC coatings, driven by sp^3^-to-sp^2^ bond transformations [223]. The level of the sp^3^-to-sp^2^ carbon bonds can be tuned by selecting the appropriate deposition technology and process conditions. These coatings are commonly deposited by reactive sputtering (Figure 2a), using DC, RF or HiPIMS power sources, depending on the desired hydrogen content, doping elements, and microstructure requirements.

For DLC coatings, an adhesion layer is mandatory to prevent delamination from the substrate. Under sliding conditions, frictional heating causes a localized transformation of sp^3^ to sp^2^ (graphitization) which leads to the formation of an ultrathin lubricious transfer layer on the counterpart material, which reduces friction by preventing direct contact between asperities. The transfer layer acts as a solid lubricant, leading to smooth sliding and reduced wear. Similarly to TMDs, DLC-based coatings have temperature limitations. The oxidation of those coatings begins at 300–400 °C, restricting their effectiveness in high-temperature environments [224].

Hydrogen-free DLC variants offer slightly better thermal stability, and improvements can be achieved through doping. This doping process is typically carried out via magnetron co-sputtering (Figure 2b) or reactive magnetron sputtering (Figure 1b), which allow the incorporation of elements such as Si, metals and N during film growth. For example, the addition of 5–20 at.% silicon stabilizes the DLC network up to 400–500 °C in air [225]. Similarly, metal and nitrogen additions and deposition of those coatings using neon (Ne) as sputtering gas have been explored to enhance thermal stability, however, the range of working temperature is still limited [226].

**Figure 6:**
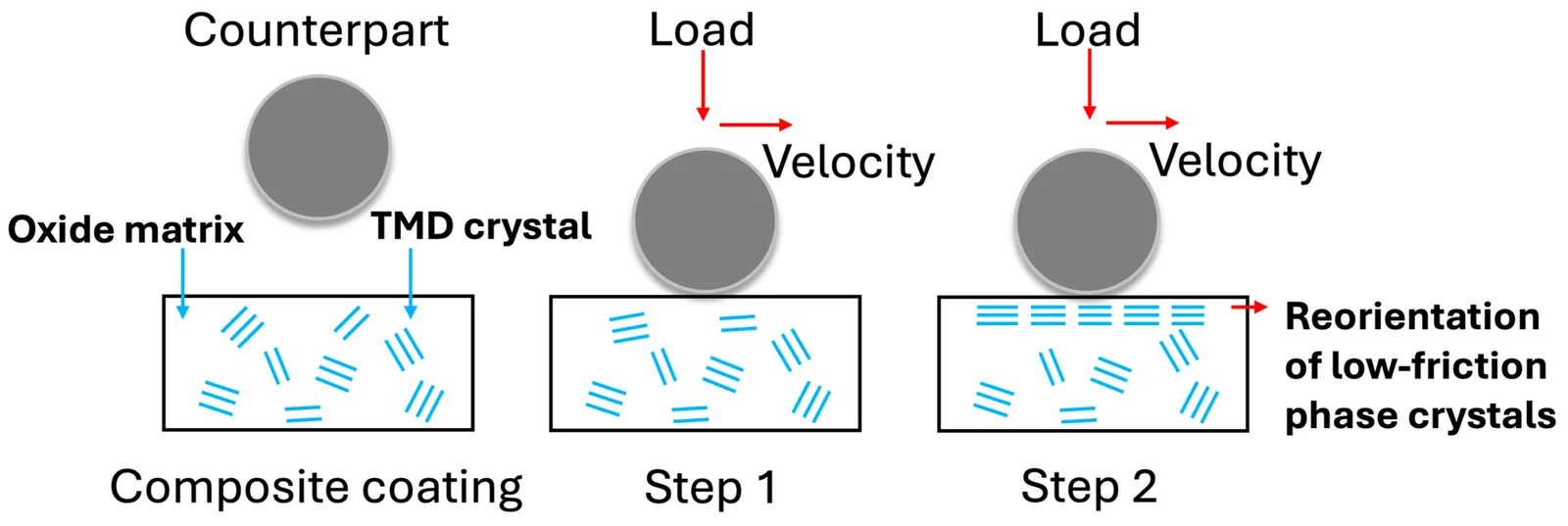
Schematic representation of a solid-lubrication coating based on an oxide matrix enclosing TMD crystals, with adaptive mechanisms based on structural transitions. The figure illustrates the alignment of the TMD material along the (0002) plane, which promotes low-friction behavior.

Incorporations of both DLC or TMD compounds into hard composite coatings have also been explored but still exhibited working temperature limitations. Hybrid approaches, deposited by co-sputtering (Figure 2b), such as tungsten carbide–tungsten disulfide–DLC (WC-WS_2_-DLC) nanocomposite coatings, further enhance adaptive tribological behavior, achieving low CoF and wear rates across various environments [227]. These modifications have led to improved tribological performance in humid air and high-temperature conditions, but their workability is still limited. The promising approach for designing coatings with adaptative mechanisms to withstand high-temperature solicitation relies on encapsulating the TMD and/or DLC materials on stable oxide matrixes (e.g., Al_2_O_3_, SiO_2_, TiO_2_, ZrO_2_) [209]. This is typically achieved using co-sputtering (Figure 2b) or sequential sputtering (Figure 2c) techniques, often combined with reactive atmospheres (Figure 1b) and substrate biasing. The oxide matrixes surrounding the low-friction materials will prevent the direct oxidation of low-friction materials by limiting exposure to oxygen and heat, whilst the low-friction phase will provide lubrication. The low-friction phase, often deposited in an amorphous state or with random orientation, will reorient parallelly to the surface of the sliding movement, providing a low-friction behavior. A schematic representation of this solution is plotted in Figure 6. This strategy ensures that the functionality of the coatings can be increased to higher-temperature (~600–1000 °C) service conditions.

### 4.2. Adaptive Mechanisms Using Metal Diffusion

**Figure 7:**
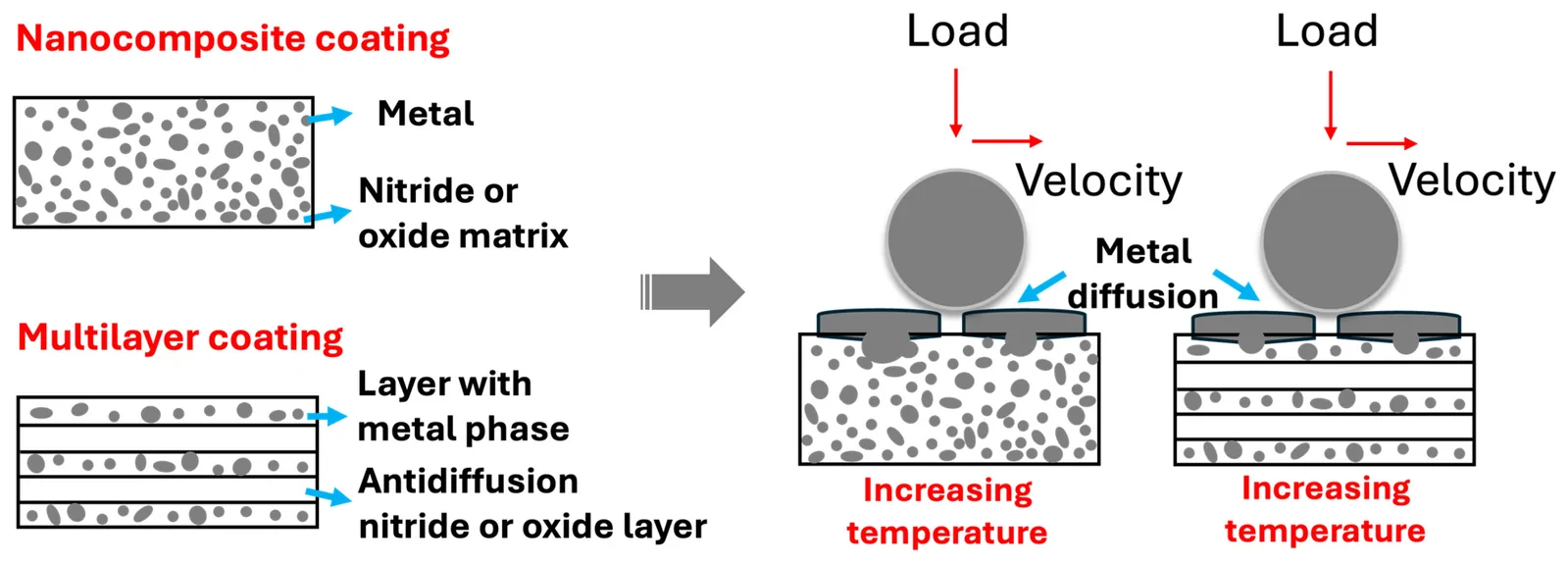
Schematic representation of solid lubrication coatings with adaptive mechanisms using metal diffusion.

The development of self-lubricating coatings utilizing metal diffusion mechanisms presents an effective solution for overcoming the temperature limitations associated with coatings based on structural transitions. The incorporation of soft metal phases provides a dynamic lubrication strategy, ensuring friction reduction and prolonged durability in extreme conditions [211]. Soft metals, characterized by their low yield strength, easily shear under mechanical stress, facilitating relative motion between surfaces with minimal resistance [228]. During tribological interactions, these metals transfer from one surface to another, forming a continuous thin film that minimizes adhesion and prevents direct metal-to-metal contact. This transfer film regenerates continuously, maintaining lubrication over extended periods. Additionally, soft metals (e.g., Ag and Au) exhibit chemical inertness and resistance to oxidation, ensuring long-term stability even under extreme temperatures, including those that induce melting. However, soft metals alone cannot be used due to their low mechanical properties. To address this limitation, self-lubricating coatings have been developed by integrating soft metals within hard, oxidation-resistant binary, ternary or quaternary films (e.g., CrN, TiSiN, CrAlN, TiAlSiN) [215,229,230,231]. These coatings are usually deposited via (reactive) co-sputtering (Figure 2b), including HiPIMS for denser microstructures, while enabling the co-deposition of the metallic component. These coatings facilitate controlled metal diffusion to the surface, forming a low-friction tribolayer. Such lubricious films have been engineered in both single and multilayer configurations. Regardless of the architecture, the introduction of these coatings significantly reduces friction and enhances wear resistance [232,233]. Nevertheless, these improvements are often short-lived due to the rapid release and depletion of the metal phase, leading to the eventual loss of the low-friction tribolayer. A promising approach to controlling the diffusion of lubricants is the incorporation of diffusion barrier layers deposited at the top the coating [234,235]. These nanometer-thick cap layers are designed to wear away gradually during service operation, ensuring a regulated release of the lubricating phase. Recent studies confirm that, to achieve long-term lubricant control, coatings should adopt a nanocomposite or multilayer architecture incorporating a stable phase with anti-diffusion properties (such as nitrides or oxides). These materials or layers must possess excellent thermal and chemical stability, as well as high mechanical strength and toughness, to sustain performance under demanding conditions. A key advantage of such coatings is their ability to self-regulate lubricant release, preventing excessive depletion at the beginning of the service life while maintaining effective lubrication over extended periods. One of the most effective nitrides for blocking soft metal diffusion is the SixN_1__−__x_ phase [212]. Though not used alone, this phase is often incorporated into composite nitrides such as TiSiN, TiAlSiN, and Mo_2_N/SiNx coatings. In a nanocomposite structure, the anti-diffusion phase encapsulates the soft metal phase, typically in the form of nanoparticles, preventing or delaying its diffusion to the surface [236]. This diffusion control can be fine-tuned by adjusting the amount and thickness of the anti-diffusion phase, as well as the position and size of the low-friction phase. In multilayer architectures, alternating layers of different materials, including one with anti-diffusion properties, are strategically designed to regulate lubricant diffusion, enhance mechanical strength, and reduce oxidation [230]. These coatings are commonly achieved through sequential sputtering (Figure 2c), in combination with substrate biasing and controlled substrate rotation to tailor layer thickness and uniformity. By adjusting the coating period, layer thicknesses, composition, and positioning of the soft metal (either within the anti-diffusion layer or in the non-anti-diffusion layer), the diffusion of the lubricating phase can be effectively controlled. Figure 7 shows a schematic representation of this advanced low-friction coating solution, highlighting the role of metal diffusion mechanisms in achieving sustained lubrication and enhanced wear resistance.

### 4.3. Adaptive Mechanisms Using Tribo-Oxidation

The mechanism beyond those adaptative coatings is very similar to the coatings utilizing metal diffusion, however, the main difference here is that an oxide layer is formed on the coating contact due to the oxidation of one or more elements [237]. This type of coating is commonly referred to as chameleon coating, as the adaptive mechanism in the contact depends on the temperature generated and on contact conditions [213,238]. In this type of coating, when the working temperature increases, tribo-oxidation occurs at the contact interface. The tribo-oxidation mechanism needs to self-regulate the formation of a lubricious oxide layer. This challenge is further compounded by the need for the surface to revert to its original state during temperature cycling. To achieve this effect, coatings must integrate materials that promote selective oxidation, forming low-friction oxides at high temperatures while preventing excessive oxidation at lower temperatures (ex., V, W, Mo, etc). This can be achieved through multilayer or nanocomposite structures, incorporating diffusion-controlling phases that regulate the release of reactive elements [239]. Such coatings are typically deposited using reactive magnetron sputtering (Figure 1b), allowing for multilayer or nanocomposite designs containing both reactive and protective elements. Those layers or matrixes must possess not only excellent thermal stability but also the ability to self-heal by regenerating the oxide layer upon repeated thermal cycling. By engineering the diffusion pathways and oxidation kinetics, a controlled chameleon-like response can be achieved, ensuring that the coating adapts dynamically to varying operational conditions while preserving its long-term functionality. Nitride and/or oxide matrixes with anti-diffusion properties should be thus used for the controlled release of the lubricious forming phases.

### 4.4. Comparative Assessment of Adaptive High-Temperature Lubrication Mechanisms

Although structural transformation, metal diffusion and tribo-oxidation are commonly presented as independent adaptive lubrication mechanisms, they should rather be regarded as complementary strategies whose suitability depends on the operating temperature, surrounding environment, coating architecture and required service life [240]. Each mechanism is activated through a different physical process and therefore exhibits distinct advantages, limitations and processing requirements. Consequently, selecting the most appropriate adaptive coating requires balancing friction reduction, oxidation resistance, mechanical integrity and long-term durability. A comparative summary of the main adaptive coating systems with examples of thin solid films developed in the last 10 years taken from the Scopus database is presented in Table 4.

Structural transformation-based coatings provide the lowest friction coefficients and exhibit the fastest adaptive response, as lubrication is generated through the reorientation or structural transformation of low shear phases directly within the tribological contact. However, their effectiveness is generally restricted to low and moderate temperatures because oxidation, humidity sensitivity and limited load-bearing capacity progressively degrade the lubricious tribolayer. Consequently, their service life is governed by the stability of the transformed phase, the amount of lubricious material available and the capability of the surrounding matrix to delay oxidation. Despite significant improvements achieved through alloying, multilayer architectures and nanocomposite designs, extending the operating temperature of these systems remains one of the principal challenges for high-temperature tribological applications.

Metal-diffusion-based coatings overcome several limitations associated with structural transformation systems by providing continuous lubricant replenishment through the thermally activated migration of a soft metallic phase towards the contact interface. As temperature increases, diffusion becomes progressively more effective, allowing lubricant release to adapt naturally to the operating conditions. Compared with structural transformation systems, these coatings generally provide a broader operating temperature window and longer service life. Nevertheless, their durability ultimately depends on maintaining a controlled lubricant reservoir, since excessive diffusion, agglomeration or depletion of the metallic phase progressively reduces lubrication efficiency. Consequently, regulating diffusion kinetics while preserving the mechanical integrity of the supporting ceramic matrix remains a fundamental design requirement [300].

Tribo-oxidation-based coatings represent the most suitable solution for severe thermal environments because lubrication is generated through the in situ formation of lubricious oxides during sliding. Unlike the previous two mechanisms, their effectiveness increases once oxidation becomes thermodynamically favorable, allowing these coatings to operate at temperatures where conventional structural-transformation systems have already lost their lubricating capability. However, their tribological performance depends strongly on oxidation kinetics, environmental atmosphere, oxide stability and the ability to continuously regenerate a mechanically stable tribofilm. Therefore, achieving a balance between sufficient oxide formation and excessive material degradation remains one of the main challenges in designing tribo-oxidation-based adaptive coatings.

From the above discussion, it is evident that none of the three adaptive mechanisms is universally superior. Instead, each mechanism occupies a different region of the temperature performance map and should be selected according to the intended operating conditions. Structural-transformation systems offer the lowest friction coefficients but possess the narrowest high-temperature stability window. Metal diffusion systems operate over a broader temperature range and provide progressive lubricant replenishment, although their lifetime is ultimately governed by lubricant depletion and diffusion control. Tribo-oxidation systems exhibit the greatest potential under severe thermal conditions, where the continuous formation of lubricious oxides can sustain low friction for prolonged periods, but their effectiveness remains highly dependent on oxidation kinetics and environmental conditions. Consequently, current research increasingly focuses on hybrid coating architectures capable of activating different lubrication mechanisms sequentially as temperature increases, thereby extending both the operating temperature window and the functional lifetime of adaptive coatings.

To overcome the relatively poor thermal stability of conventional structural transformation systems, recent research has increasingly focused on microstructural engineering rather than simply modifying coating composition. The principal strategies include elemental doping, nanocomposite and multilayer architectures, encapsulation of lubricating phases within oxidation-resistant matrixes, diffusion control barriers and graded interfaces. These approaches aim to delay oxidation, improve mechanical integrity, regulate lubricant release and preserve adaptive lubrication over progressively wider temperature ranges.

Elemental doping remains one of the most effective approaches for improving thermal stability because it modifies the microstructure, oxidation resistance and mechanical properties of the lubricating phase without fundamentally altering the adaptive lubrication mechanism. Likewise, nanocomposite and multilayer architectures enhance load-bearing capacity while reducing oxygen transport or metallic element diffusion through nanoscale interfaces, thereby delaying degradation of the lubricating phase. Encapsulation within thermally stable oxide matrixes provides an additional level of protection by isolating the lubricating phase from direct oxidation while allowing progressive activation during sliding. Finally, diffusion barriers and graded interfaces regulate lubricant transport, improve adhesion and reduce thermal and mechanical incompatibilities between adjacent layers, contributing to longer coating lifetime under severe operating conditions.

The recent developments clearly demonstrate that the high-temperature limitations of conventional adaptive coatings cannot be overcome through a single modification strategy. Instead, the most effective coating architectures integrate multiple stabilization mechanisms, including elemental doping, nanocomposite design, oxide encapsulation, diffusion barriers and graded interfaces. Increasingly, multifunctional adaptive coatings are being designed so that different lubrication mechanisms become sequentially active as temperature increases, combining the low-friction behavior characteristic of structural-transformation systems at low temperatures with the lubricant replenishment associated with metal diffusion and the excellent high-temperature stability provided by tribo-oxidation. This sequential activation represents one of the most promising directions for the development of next-generation adaptive self-lubricating coatings capable of operating over an exceptionally wide temperature range.

## 5. Section C—The Emergence of Thin-Film Sensors as a Solution for High-Temperature Measurement in Protective Coatings

In metal cutting operations, monitoring the tool–chip interface temperature is crucial for evaluating machining conditions. This temperature directly correlates with key factors such as tool wear, dimensional stability, surface integrity, and machine downtime. However, obtaining direct and accurate temperature measurements in this highly localized region presents significant technical challenges due to limited accessibility, small measurement zones, and mechanical stresses.

Conventional temperature sensing solutions, such as thermocouples, typically require substantial modifications to the cutting tool, workpiece, or surrounding environment. These modifications can compromise tool performance, limit material selection, and restrict machining parameters [301,302]. As a result, there is a need for innovative temperature monitoring techniques that minimize disruption to the machining setup.

**Figure 8:**
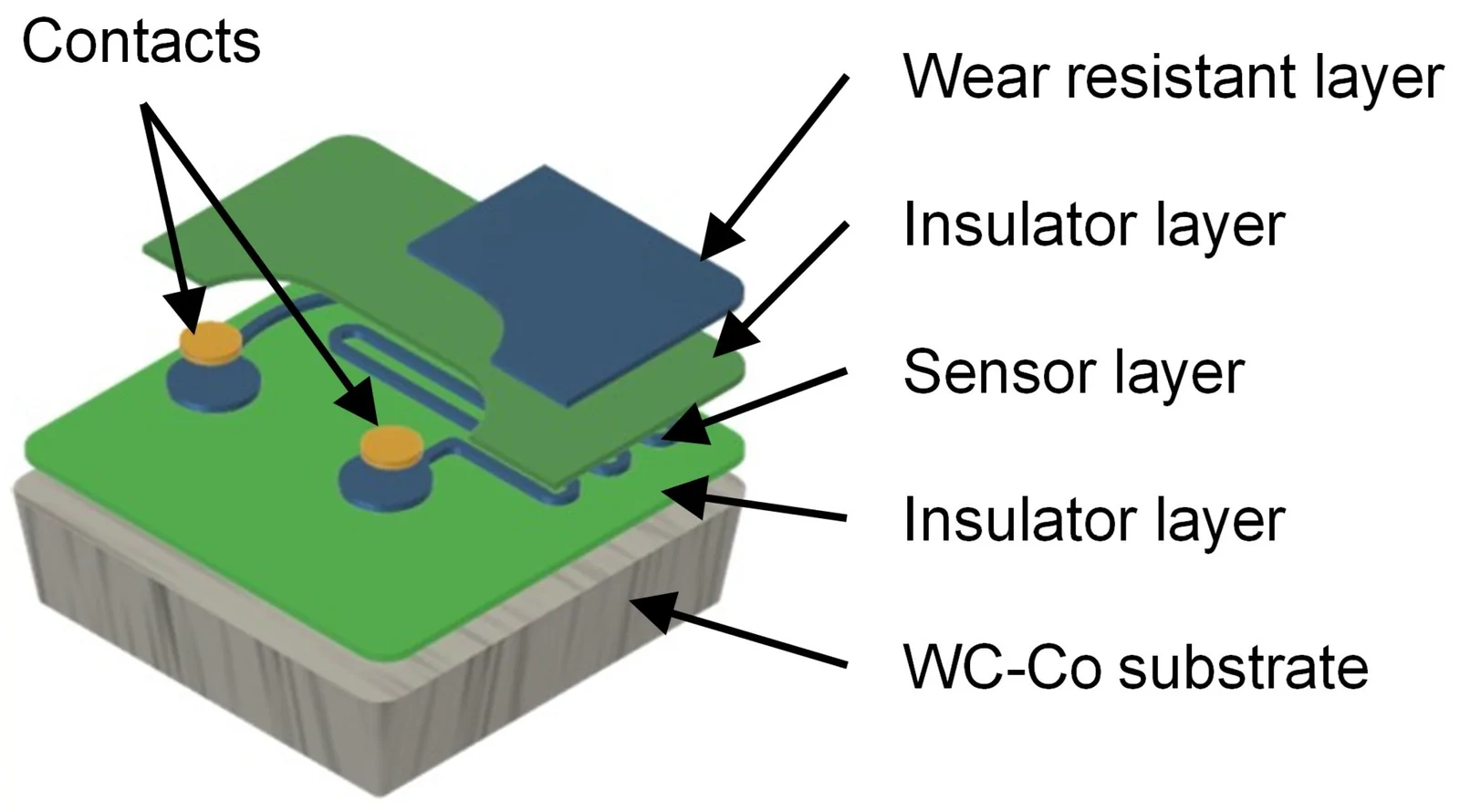
Schematic of a multilayer architecture for electrical insulation and wear protection of thin-film sensors. Adapted from [304].

To address these challenges, thin-film sensor coatings deposited by PVD techniques—particularly sputtering—have emerged as promising alternatives for sensor integration. Since the 1980s, sputtering technologies have been explored to embed sensors directly at critical tool locations, enabling real-time, localized monitoring during machining operations [303]. Thin-film sensors offer several advantages, including minimal impact on the tool’s geometry, fast response times due to their small thermal mass, and localized temperature detection. Despite their potential, integrating thin-film sensors presents significant engineering challenges, primarily related to the requirement for complex multilayer architectures. These multilayers are essential to ensure electrical insulation and protection from wear and thermal loads during cutting operations (Figure 8).

Sputtering technology plays a fundamental role in sensor integration mainly due to its exceptional versatility in depositing a wide range of materials, including metals, semiconductors, ceramics, and insulating oxides or nitrides. This versatility facilitates the fabrication of complex multilayer sensor architectures—such as those illustrated in Figure 8—in single-chamber sputtering systems equipped with multiple material targets (Figure 2). Additionally, sputtering’s adaptability allows fine-tuning of critical functional properties, such as electrical conductivity, thermal sensitivity, and wear resistance, which are vital for sensors operating under demanding cutting conditions. Consequently, sputtering has become indispensable for advancing sensor design and enabling their robust deployment in industrial machining applications.

### 5.1. Thin-Film Thermocouples

**Figure 9:**
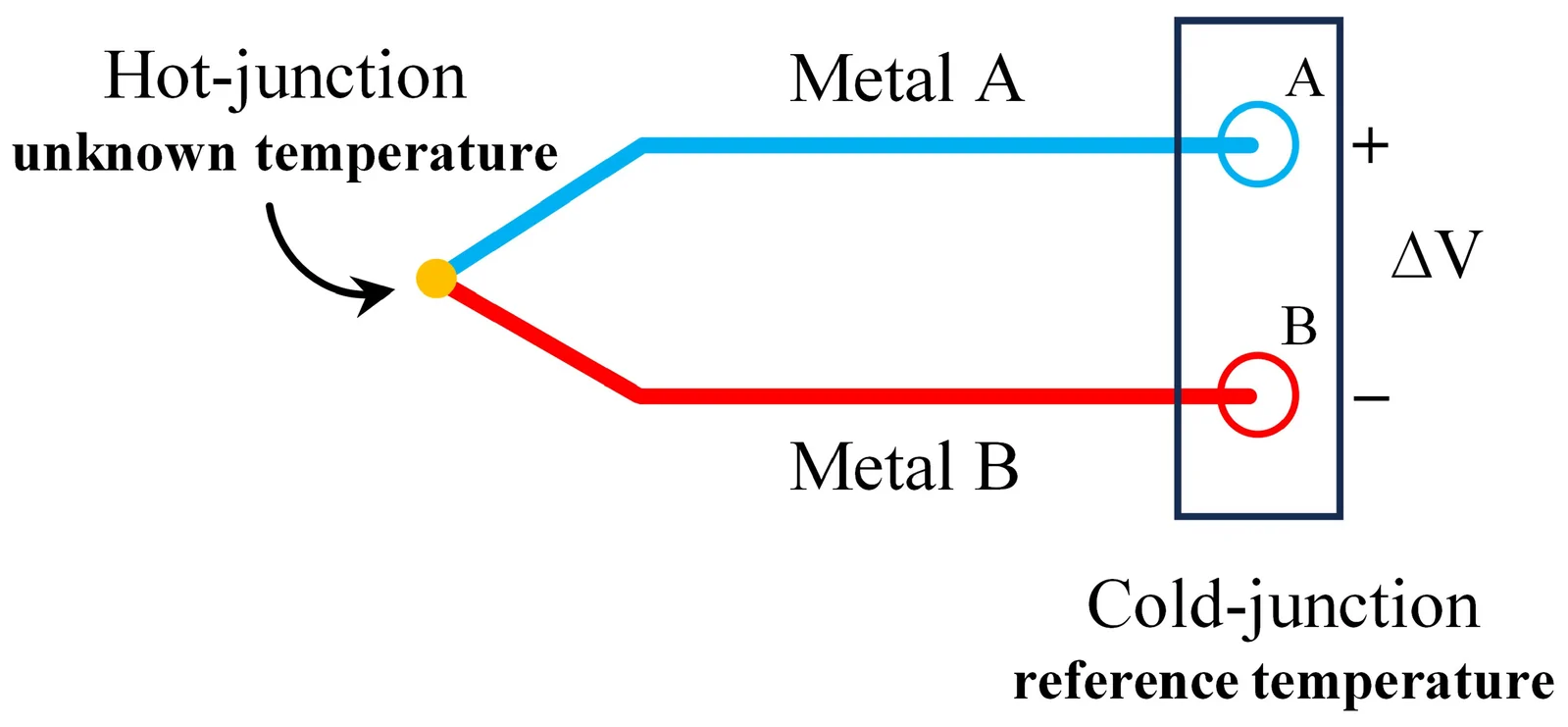
Simplified schematic of a thermocouple illustrating the necessity for cold-junction compensation.

Among the various sensing mechanisms explored, the thermoelectric effect—more specifically, the Seebeck effect—remains the most widely used principle, with thin-film thermocouples (TFTCs) being the dominant approach for surface temperature sensing [305]. The Seebeck effect describes the voltage (V) generated between two dissimilar conductors as a function of the temperature difference (∆T) between the hot (measurement) and cold (reference) junctions (Figure 9). However, practical implementation requires addressing a critical but often overlooked challenge: cold-junction compensation. As illustrated in Figure 9, when the thermocouple leads are connected to a measuring device (points A and B), additional unintended junctions are created. Accurate temperature determination at the hot junction depends on knowing the temperature at the cold junction, which is particularly complex in cutting tools due to spatial limitation and thermal gradients.

Many studies have sought to simplify sensor integration by utilizing electrically insulating substrates to minimize multilayer complexity. Alumina (Al_2_O_3_) and polycrystalline cubic boron nitride (PCBN) cutting inserts are particularly attractive choices due to their inherent electrical insulation properties. In one study [306], K-type TFTCs (Ni/Ni-Cr junctions) were integrated onto Al_2_O_3_ inserts using magnetron sputtering, combined with photolithography to achieve high-resolution sensor patterning. To ensure electrical isolation and wear protection, additional layers of hafnium oxide (HfO_2_) and TiN were deposited. This approach enabled temperature measurements of up to 600 °C during the dry turning of aluminum alloy (A6061). In another study [307], Al_2_O_3_ inserts featuring microgrooves machined into the tool’s rake face were utilized, where Cu/Ni thermocouple pairs were deposited. These TFTCs were then covered with a silicon nitride (Si_3_N_4_) layer to provide additional wear protection and electrical insulation. Although these sensors successfully measured temperatures up to 500 °C during the turning of aluminum alloy (A5056), the study did not include essential details regarding the electrical connections.

Comparable strategies have been applied using PCBN substrates. One study [308] reported the fabrication of C-type TFTCs (tungsten–rhenium, W-Re) on PCBN cutting inserts via an intricate combination of photolithography, evaporation, sputtering, and diffusion bonding. Although these sensors effectively recorded temperatures up to 230 °C during the machining of Ti_6_Al_4_V, the complex fabrication process raised concerns about reproducibility and practical implementation. In a related study [309], similar C-type TFTCs were sputter-deposited onto PCBN inserts, insulated by a thermally evaporated Al_2_O_3_ layer. Although the sensor exhibited good linearity, the calibration was limited to relatively low temperatures (125 °C), restricting its relevance to high-temperature machining conditions. Another study [310] focused on N-type (NiCr/NiSi) TFTCs deposited on PCBN inserts reported sensor calibration up to 1000 °C. However, in actual machining tests, these sensors recorded unexpectedly low temperatures (~60 °C). This discrepancy suggested suboptimal sensor placement or inadequate protection, limiting the effectiveness of the approach. In another study focusing on non-conductive substrates [311], Ni-NiCr thermocouple pairs were deposited onto Si_3_N_4_ inserts, allowing for temperature measurements up to 300 °C during the machining of titanium alloy (Ti_10_C_2_Fe_3_Al). However, misalignment issues and poor adhesion to the substrate highlighted practical challenges in sensor integration.

Sensor integration has also been attempted on conventional WC-based cutting inserts, requiring additional electrical insulating layers. These efforts typically involved embedding sensors within microgrooves machined into the inserts’ rake faces, using layers for electrical insulation and wear protection such as Si_3_N_4_ [312,313] and Al_2_O_3_ [314]. Standard K-type (alumel/chromel) TFTCs embedded in these configurations demonstrated linear responses and sensitivities comparable to standard thermocouples during the dry cutting of titanium alloy (Ti_6_Al_4_V). However, discrepancies emerged across similar studies conducted under comparable machining conditions, raising concerns about reproducibility and consistency [312,313,314,315]. In another study [316], N-type TFTCs were embedded within silicon oxide (SiO_2_) insulating layers on WC inserts, successfully recording stable sensor outputs up to 600 °C during the dry turning of Ti_6_Al_4_V alloy. However, direct comparisons with earlier literature were hindered by incomplete disclosure of machining parameters, such as cutting speed.

More comprehensive multilayer sensor architectures were developed resembling the schematic depicted in Figure 8, incorporating and integrating insulation layers, K-type TFTCs, and wear protective top layers. For instance, multilayer structures, including Al_2_O_3_ for insulation and TiAlN for wear protection, have been systematically tested during machining operations involving various steel alloys (AISI 12L14 [317], AISI 4130 [318], and AISI 4140 [319,320]). These sensors consistently demonstrated stable temperature measurements (up to approximately 550 °C), showcasing effective insulation and wear protection, while also confirming the efficiency of conventional cooling strategies [320]. Despite these promising results, conducting a comparative analysis to validate the consistency of these multilayer systems remains challenging, primarily due to variations in cutting parameters, experimental configurations, and tool layouts across different studies.

Despite significant progress in integrating TFTCs within cutting tools, several recurring limitations continue to hinder their practical adoption in industrial settings. One of the most critical challenges is the lack of clear and consistent methods for cold-junction compensation, which introduces uncertainties in measurement accuracy and reliability [306,307,308,309,319]. While some studies acknowledge the importance of this aspect [310,319], they often fail to provide detailed descriptions or validation methods for cold-junction compensation. Another major barrier is the complexity of sensor fabrication. Many approaches involve multiple deposition and patterning steps, which complicate consistent and scalable implementation [306,307,309,312,313,314]. This complexity is further compounded by challenges related to electrical connections. For instance, the common reliance on silver paste or protective polymeric coatings (e.g., Kapton^®^ tape) can inadvertently introduce unintended thermal junctions, potentially affecting temperature measurements [308,309,312,316,317]. Additionally, sensor reliability can be compromised by exposed wiring, insufficient insulation, weak electrical connections, and spatial constraints, which may collectively restrict the applicability of these approaches under typical industrial machining parameters.

### 5.2. Thin-Film Thermoresistive Sensors

Thermoresistive sensors primarily fall into two categories: negative temperature coefficient (NTC) thermistors and positive temperature coefficient of resistance (TCR) sensors, commonly referred to as resistance temperature detectors (RTDs). NTC thermistors exhibit a non-linear decrease in electrical resistance as temperature rises, a characteristic resulting from their semiconductor behavior, while RTDs typically exhibit a linear increase in resistance with temperature due to thermal oscillations of ions, hindering electron mobility [321]. Among thermoresistive sensors, semiconductor-based NTC thermistors have gained considerable attention owing to their exceptional sensitivity (quantified by the thermal constant, β), rapid response times, cost-effectiveness, and high electrical resistivity [322]. The inherently high resistivity of NTC thermistors is particularly advantageous for remote or complex measurement setups, significantly reducing the influence of lead-wire resistance and thereby simplifying instrumentation to basic two-point voltage divider circuits [323]. In contrast, RTDs, typically composed of pure metals such as platinum, offer more linear and predictable resistance–temperature relationships. This linearity makes RTDs particularly suitable for precise temperature measurements and simplifies sensor calibration, albeit at the cost of lower sensitivity and lower intrinsic resistivity.

**Figure 10:**
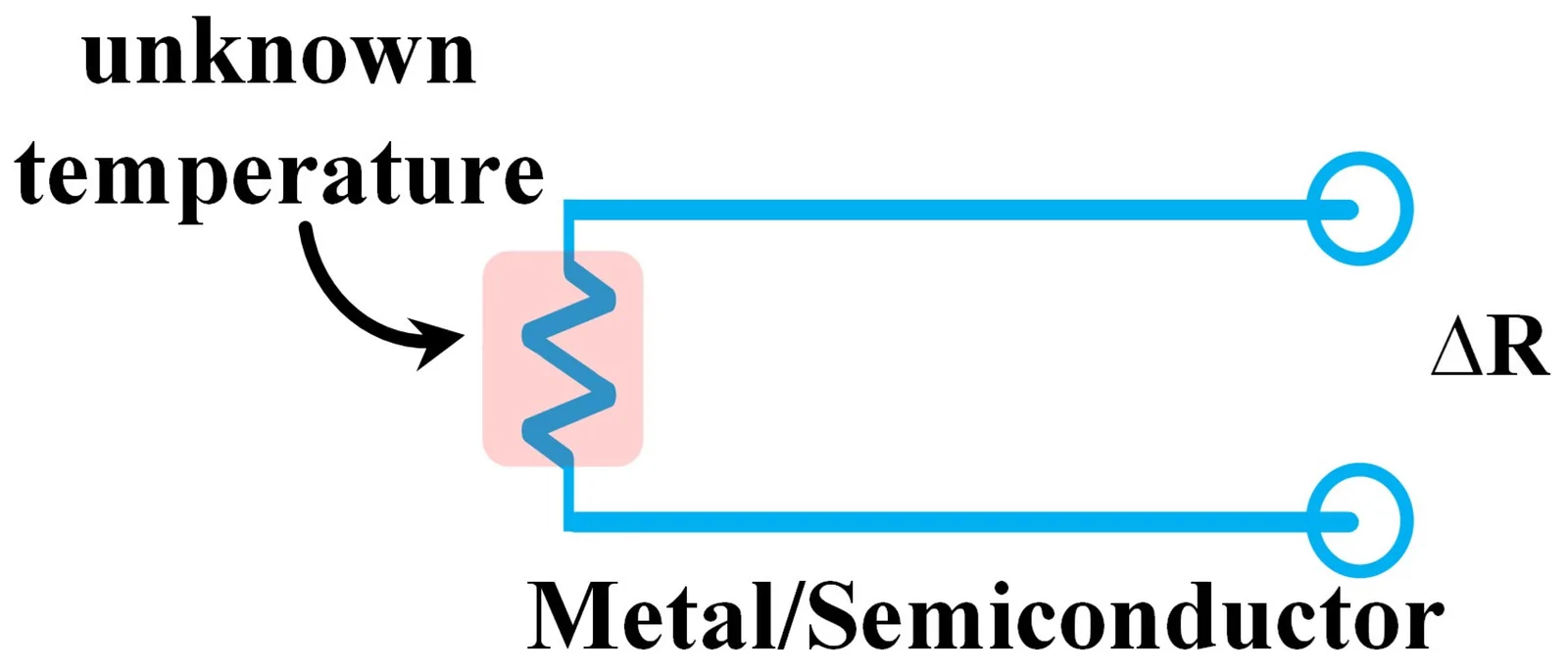
Simplified schematic of a thermoresistive sensor.

When implemented as thin-film sensors, thermoresistive detectors present several practical advantages over TFTCs, including significantly simpler sensor integration and signal acquisition. Thermoresistive sensors rely solely on measuring electrical resistance variations as a function of temperature (Figure 10), circumventing the need for additional interfaces required by TFTCs, which depend on two dissimilar materials to generate the thermoelectric (Seebeck) effect. Importantly, resistive sensors eliminate the challenges associated with cold-junction compensation inherent to TFTCs, thereby simplifying calibration and improving measurement reliability. Nonetheless, thermoresistive sensors are not without limitations. NTC thermistors, despite their superior sensitivity, often exhibit non-linear behavior and limited operational temperature ranges, typically constrained below 300 °C. Conversely, RTDs overcome many of these limitations by providing linear resistance–temperature characteristics but also have limited temperature ranges. Their comparatively low electrical resistivity typically necessitates a four-point measurement configuration to eliminate lead-wire resistance effects, thus introducing additional complexity into the measurement setup.

Investigations have focused on thin-film resistive sensors for measuring cutting temperatures in machining applications. In one example, a multilayer chromium-based thermoresistive sensor, deposited by PVD, was developed to monitor cutting temperatures during the turning of AISI 4140 steel [324]. In this approach, a Cr sensor was encapsulated between two Al_2_O_3_ layers serving as electrical insulation and wear protection. The sensor patterning was accomplished through photolithography, and sensor calibration demonstrated stable and predictable resistance–temperature characteristics up to 170 °C, enabling temperature measurements nearing 250 °C during machining. In a subsequent study [325], the same Cr-based resistive sensor was further developed for temperature compensation of DLC and manganin-based piezoresistive sensors designed to measure cutting forces in the same AISI 4140 steel. In this setup, the temperature sensor was deposited on a cutting tool including an Al_2_O_3_ layer for wear protection and chip-break geometry. The geometric constraints promoted by the chip-break feature prevented the sensor placement closer to the tool–chip interface to avoid electrical failures due to interruptions of the conducting paths. Consequently, this drawback prevented the sensors from achieving steady-state temperature conditions within short measurement intervals. Building on these developments, further research [326] focused on validating numerical simulation models of machining temperature distribution by improving sensor calibration techniques, including a specialized test rig capable of calibration up to 400 °C. While the study established correlations between experimental measurements and simulation results during the orthogonal cutting of AISI 4140 steel, it did not directly compare these results with previous experimental data on the same workpiece material, which could have strengthened validation.

**Figure 11:**
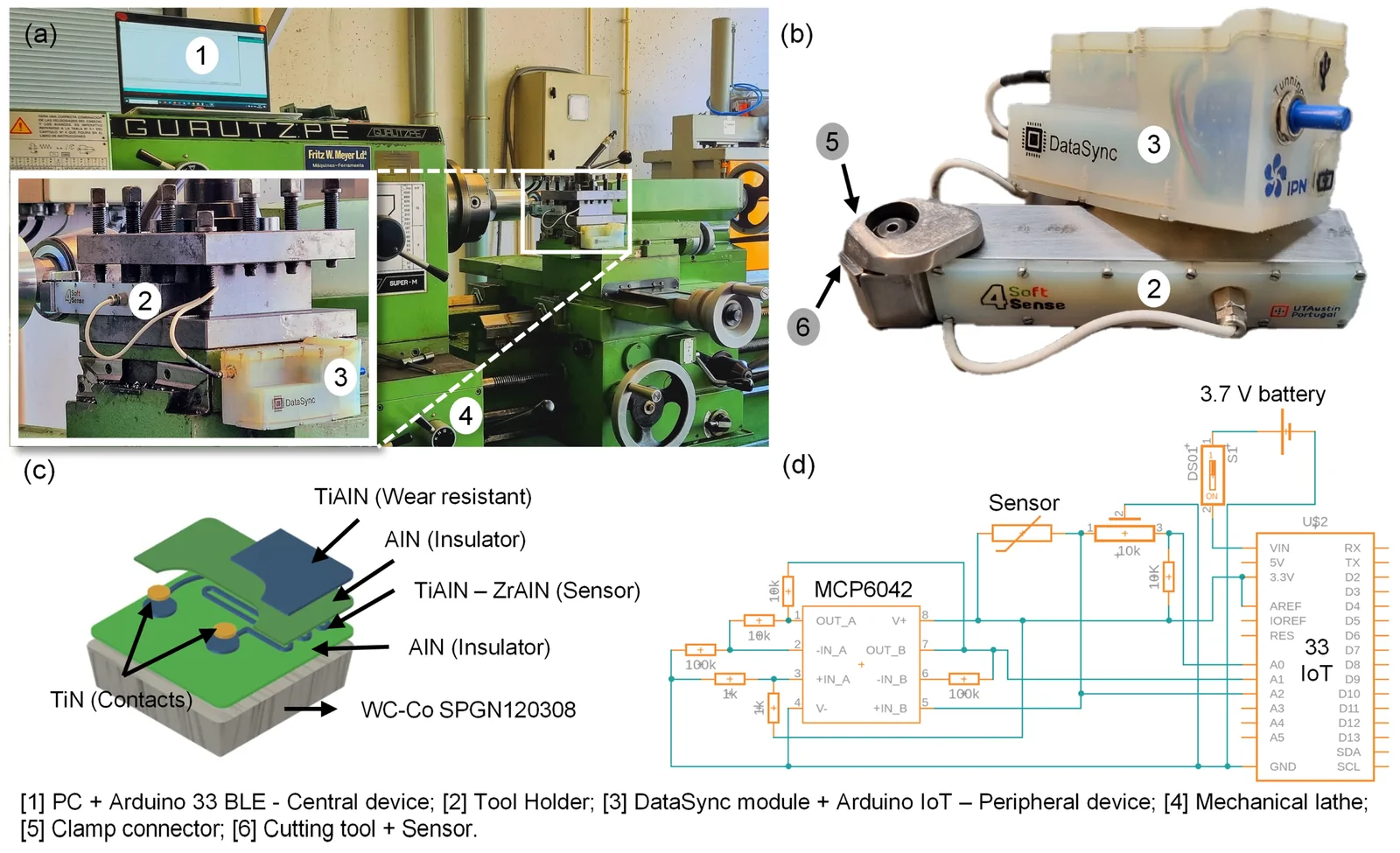
Experimental configuration and diagram illustrating the temperature measurement setup in cutting tools using TiAlN and ZrAlN thin-film thermistors: (**a**) cutting experiments apparatus; (**b**) tool holder and signal conditioning module; (**c**) multilayer architecture with integrated sensor; (**d**) electrical schematic for data acquisition–transmission. Reprinted from [304].

Another area of research focuses on the integration of semiconductor-based thin-film NTC thermistors into cutting tools for direct temperature monitoring during machining processes. Initial investigations have explored the feasibility of adapting wear-resistant coating materials, such as TiAlN [327,328] and zirconium aluminum nitride (ZrAlN) [329,330], as temperature-sensitive layers within multilayer nitride coatings. These coatings were deposited via magnetron sputtering (Figure 2c), following the multilayer architecture illustrated in Figure 8. Both sensor materials exhibited stable semiconductor behavior and reliable temperature sensitivity up to 750 °C. However, when integrated within the multilayer coating, their operational temperature range decreased to 400 °C. Despite these promising characteristics, both materials exhibited relatively low sensitivity levels, with TiAlN showing a sensitivity factor of approximately β ≈ 100 K [328], while ZrAlN displayed a higher sensitivity of β ≈ 850 K [330]. Subsequent studies have demonstrated the practical applicability of these multilayer sensor systems under realistic machining conditions involving the titanium alloy Ti_6_Al_4_V [304]. While the embedded sensors remained functional at temperatures as high as 750 °C, reliable calibration was limited to approximately 400 °C. Nevertheless, these investigations provided proof-of-concept validation by successfully measuring cutting temperatures under varied machining parameters. To enhance usability, the study introduced a novel plug-and-play connector for signal retrieval, accompanied by wireless data transmission capabilities, thus advancing sensor integration (Figure 11). However, the study acknowledged that the distance of the sensors from the cutting edge could prevent the capture of peak temperatures, as the maximum recorded temperature was only 210 °C.

### 5.3. Comparative Assessment, Measurement Variability, and Implementation Outlook

A meaningful comparison of thin-film temperature sensors requires consideration not only of the sensing principle but also of the complete sensor architecture, fabrication route, calibration procedure, signal acquisition system, sensor position, and integrity of the insulating and protective layers. These factors complicate comparisons not only between thermocouples and thermoresistive sensors but also between studies employing the same sensing principle.

Table 5 summarizes the main studies reporting temperature measurements during the machining of aluminum, titanium, and steel alloys, together with the main cutting conditions, sensor architecture, fabrication steps, calibration range, and maximum temperature measured.

Thin-film thermocouples remain the most extensively investigated approach for temperature measurement in cutting tools. Their principal advantages include a small sensing junction, rapid thermal response, and the availability of several material combinations for different temperature ranges. Calibration temperatures of up to 1000 °C have been reported, although most machining experiments were conducted at substantially lower temperatures [306,307,311,312,313,314,316,317,319,320]. Their integration nevertheless requires two dissimilar conductive films and a well-defined measurement junction. Furthermore, the low thermoelectric output generally requires signal amplification or dedicated instrumentation. Reliable measurement also depends on cold-junction compensation and on avoiding unintended thermoelectric junctions at the transitions between the sensing films, wires, conductive adhesives, and connectors. Thermoresistive sensors use a single temperature-sensitive element and therefore avoid cold-junction compensation. Metallic films usually provide an approximately linear resistance–temperature response, but their relatively low resistance makes the measurement more sensitive to lead and contact resistances, potentially requiring three- or four-wire configurations [324,325,326]. Semiconductor thermistors exhibit substantially higher electrical resistance, reducing the relative contribution of connecting wires and enabling simpler two-wire acquisition systems [304,327,328,329,330]. However, their non-linear response requires suitable calibration and may be affected by self-heating and signal hysteresis.

The calibration range, thermal stability of the sensing material, and operating range of the complete device should also be distinguished. For instance, TiAlN- and ZrAlN-based films retained semiconductor behavior up to 750 °C, whereas reliable calibration of the complete multilayer sensor architecture was limited to approximately 400 °C [304,327,328,329,330]. This difference shows that the sensing film is not necessarily the component controlling the operating limit. Electrical insulation and conducting-path continuity may instead determine whether the sensor remains functional during machining. Similarly, calibration at high temperature does not demonstrate that the integrated sensor can withstand prolonged or repeated cutting operations. Most studies consist of relatively short proof-of-concept experiments and provide limited information on cumulative machining time, repeated cutting cycles, or sensor survival rate. Long-duration testing under representative industrial conditions is therefore required to determine whether the reported architectures retain their performance throughout the service life of the cutting insert.

A rigorous comparison of measurement accuracy is currently difficult because few studies provide uncertainty analysis or validation against an independent temperature measurement technique. The reported values should therefore not be interpreted as direct evidence that one sensing principle is more accurate than another. At present, both thermocouple and thermoresistive architectures appear more suitable for identifying temperature variations caused by changes in machining conditions than for determining the absolute temperature at the tool–chip interface with a well-established measurement uncertainty.

The variation in the maximum temperatures reported in Table 5 reflects differences in workpiece machinability, cutting conditions, tool geometry, insert material, test duration, and sensor architecture. Even for the same workpiece material and apparently similar cutting parameters, the measured temperature may differ because the sensors are not necessarily located at equivalent positions. Sensors placed close to the cutting edge can detect more localized thermal events, whereas sensors positioned farther away may provide a less representative measurement of the interface temperature. Although increasing the sensor-to-edge distance may facilitate patterning, electrical connection, and protection against mechanical damage, it reduces spatial resolution and the likelihood of detecting the maximum interface temperature. Several studies have highlighted sensor positioning as a key factor, since the distance from the cutting edge may influence the representativeness of the measured temperature [304,306,307,313].

Calibration introduces an additional source of uncertainty when the temperature measured during machining approaches or exceeds the experimentally validated calibration range. Under these conditions, conversion of the electrical signal into temperature may require extrapolation. This issue is evident in some studies summarized in Table 5, where the maximum reported machining temperature exceeded the stated calibration range. Such results may still demonstrate sensor functionality and sensitivity to machining conditions, but the corresponding absolute temperature values should be interpreted cautiously. From an implementation perspective, no architecture has yet demonstrated all the requirements for widespread industrial adoption. The most promising development route is not necessarily associated with the sensor exhibiting the highest calibration temperature or the highest temperature measured during machining. Architectures requiring microgrooves, diffusion bonding, several deposition systems, extensive photolithographic processing, or numerous electrical interfaces may present significant challenges in terms of reproducibility and scale-up. Greater importance should be attributed to achieving an appropriate balance between sensitivity, calibration range, wear resistance, reproducible fabrication, and signal conditioning.

## 6. Conclusions

Protective coatings deposited by PVD techniques, particularly sputtering, have become integral to advancing material performance across a wide range of industrial applications. The versatility, precision, and environmentally friendly nature of sputtering processes have established them as key technologies for enhancing the durability, aesthetics, and functionality of coated surfaces. This review has focused on three major application areas: decorative coatings, high-temperature coatings, and temperature monitoring sensors. Despite their distinct end-use requirements, these applications share common challenges related to material stability, integration strategies, and the need for enhanced performance under demanding conditions.

### 6.1. Decorative Applications

Sputtering technology has transformed the field of decorative coatings by offering robust, aesthetically versatile, and sustainable alternatives to conventional surface treatments. The ability to tailor the appearance of coatings through both intrinsic and structural coloration mechanisms has significantly broadened their applicability. Intrinsic coloration, achieved by selecting appropriate transition metal nitrides and carbonitrides, allows for the creation of coatings with vibrant, metallic hues such as gold, bronze, silver and bluish-purple. These coatings not only provide visual appeal but also deliver superior hardness, wear resistance, and thermal stability, making them particularly suitable for automotive components, jewellery, watches, and architectural hardware.

Structural coloration, on the other hand, exploits nanoscale design to achieve vivid colors without the need for pigments. Coating architectures such as multilayer thin-films—relying on optical interference—and nanocomposites embedded with metal nanoparticles—exploiting localized light absorption and scattering—allow for precise tuning of optical properties. This strategy not only enhances color fidelity but also enables the integration of additional functionalities, such as anti-bacterial activity or plasmonic sensing.

With continued progress in materials science and nanotechnology, the next generation of sputter-deposited decorative coatings is expected to merge aesthetic appeal with multifunctional performance, meeting the demands of high-end applications where both visual quality and long-term durability are essential. However, current studies reveal that the incorporation of additional functionalities (e.g., anti-microbial activity) often compromises the target coloration, highlighting the need for improved coating design strategies.

### 6.2. High-Temperature Applications

The development of coatings for high-temperature applications has been driven by the demand for low-friction, wear-resistant surfaces that maintain functionality in harsh thermal environments. Self-lubricating coatings, particularly those based on transition metal dichalcogenides (TMDs) and diamond-like carbon (DLC), have emerged as promising candidates. These coatings often rely on structural transformations or metal diffusion mechanisms to reduce friction; however, maintaining stability above 300–400 °C remains a significant challenge due to oxidation and mechanical degradation.

Advanced approaches have focused on encapsulating lubricating phases within oxide matrixes to enhance their thermal endurance. Additionally, coatings that utilize metal diffusion mechanisms, such as Ag migration to the surface under stress, have shown potential but face issues with lubricant depletion. Integrating tribo-oxidation mechanisms has proven effective in forming lubricious oxides at high temperatures, but precise tuning is required to maintain consistent performance. Moving forward, a balanced approach that combines friction reduction, structural integrity, and adaptive responses to thermal cycling will be essential for developing coatings capable of enduring prolonged high-temperature exposure.

### 6.3. Temperature Monitoring Applications

The integration of thin-film temperature sensors into cutting tools has demonstrated the feasibility of localized, real-time temperature monitoring during machining. Thin-film thermocouples remain the most extensively investigated and established approach, offering rapid response and broad material flexibility. However, their implementation requires two dissimilar sensing films, cold-junction compensation, low-noise signal conditioning. Thermoresistive sensors provide a simpler sensing architecture and avoid cold-junction compensation. Metallic resistive films offer an approximately linear response, whereas high-resistivity semiconductor thermistors enable simpler two-wire acquisition. Despite these advances, no sensor architecture has yet demonstrated all the requirements for widespread industrial adoption. The main limitations are associated with the complete multilayer stack, including electrical insulation, contact stability, durability, electrical path continuity, and sensor positioning. Sensors located farther from the cutting edge are easier to protect, but their measurements may be less representative of the temperature at the tool–chip interface.

Comparison between published results also remains difficult because relatively few studies report uncertainty measurements, independent validation, or long-term performance under repeated cutting operations. Calibration range, sensing-material stability, and the operating range of the integrated device should therefore be treated as distinct parameters. Accordingly, the highest calibration temperature or maximum machining temperature does not necessarily indicate the most reliable or industrially relevant sensor architecture. Architectures requiring extensive micromachining, multiple deposition systems, or numerous assembly stages are likely to remain less attractive for industrial scale-up than designs based on conventional inserts and simplified fabrication routes. Recent developments in modular connectivity and wireless data transmission have improved signal communication, while deposited insulating layers have extended sensor integration from intrinsically insulating tool materials to standard conductive WC inserts. Nevertheless, further efforts should focus on long-term durability, reliable signal acquisition, and validation across a broader range of machining conditions, tool geometries, and workpiece materials.

### 6.4. Broader Implications and Future Perspectives

While each application area addressed in this review exhibits unique challenges and requirements, the fundamental goal of PVD coating technology remains the same: to improve material performance through advanced surface engineering. The rapid evolution of PVD methods, especially sputtering, offers exciting prospects for creating coatings that are not only more durable and functional but also more adaptable to specific industrial needs.

Future research should aim to develop coatings with hybrid functionalities that address multiple performance criteria simultaneously. For decorative applications, combining aesthetic appeal with anti-fingerprint and anti-microbial properties can expand the use of PVD coatings in consumer goods. In high-temperature contexts, advancing coatings that dynamically adapt their frictional properties while maintaining structural integrity will improve the efficiency and lifespan of critical components. For temperature monitoring, future work should prioritize multilayer sensor architectures compatible with standard cutting inserts and tool holders, reliable electrical connections, and wireless signal-conditioning systems. Long-duration cutting tests and sensor-to-sensor reproducibility should also be systematically assessed.

Beyond application-specific developments, future advances in PVD technology will increasingly rely on digital approaches and data-driven methodologies. Artificial intelligence and machine learning algorithms, combined with materials informatics, are expected to accelerate the discovery and optimization of coating compositions, architectures, and deposition parameters by establishing relationships between processing conditions, microstructure, and functional performance. Such approaches may enable more efficient and predictive coating design, reducing the reliance on extensive experimental trial-and-error procedures. Furthermore, the development of multifunctional coatings capable of operating under extreme conditions, including high temperatures, severe mechanical loads, and chemically aggressive environments, represents another important research direction. Finally, the transition from laboratory-scale demonstrations to industrial implementation remains a critical challenge, requiring the development of scalable deposition processes, reproducible manufacturing strategies, and validation under realistic operating conditions.

Addressing the current limitations of PVD coatings requires continued interdisciplinary efforts, bringing together material science, surface engineering, computational methods, and industrial manufacturing. By combining advanced coating design, intelligent coating architectures, and scalable production approaches, PVD technology can continue to meet the increasingly complex demands of modern industry, ensuring reliable, multifunctional, and sustainable surface solutions.

## Figures and Tables

**Table 1:** Comparison of the main sputter deposition routes, highlighting their advantages, limitations, representative coating architectures, and typical applications.

Deposition Strategy	Main Advantages	Main Limitations	Typical Coating Architectures	Representative Applications
**Conventional sputtering**	Simple, scalable, uniform, dense coatings	Limited compositional flexibility	Monolithic films	Protective, optics, decorative, electronics, semiconductor interconnects
**Co-sputtering**	Excellent composition control	Requires multiple power supplies	Solid solutions, nanocomposites	Catalysts, photonics, plasmonics, decorative, HEA, photovoltaics, advanced metallurgy
**Sequential sputtering**	Sharp interfaces	Lower throughput	Multilayers, superlattices	Protective, tribology, optics, decorative, magnetoresistance, diffusion barriers, sensors
**GLAD**	Highly porous, anisotropic structures	Complex deposition, line-of-sight	Helices, zig-zags, columns	Sensors, photonics, plasmonics, superhydrophobic/self-cleaning
**Reactive** **sputtering**	Oxides, nitrides, carbides	Target poisoning, hysteresis	Compound coatings	Protective, photonics, plasmonics, decorative, optoelectronics, sensors
**PGC**	Nanoparticle synthesis	Complex equipment	Nanocomposites	Catalysts, sensors, plasmonics, quantum dots, biomedical, nanomedicine

**Table 2:** Colors and properties of decorative protective PVD coatings. The background colors are only an approximate visual reference.

System	Colors	Properties	Max. Service Temp. (°C)
**Ti_x_N_1_** **_−_** **_x_**	**Gold**	High hardness and toughness, biocompatible and non-toxic, reduced friction, compatible with acids/bases/solvents, low anti-fouling performance	600
	**Bronze**		
	**Brown**		
**Zr_x_N_1−x_**	**Pale gold**	High hardness/toughness, good wear resistance, excellent corrosion resistance, biocompatible, low anti-fouling performance	550
	**Champagne gold**		
	**Brass**		
	**Nickel**		
**Cr_2_N, Cr_x_N_1−x_**	**Silver**	High hardness/toughness, reduced friction, resistant to sliding and impact wear, excellent resistance to corrosion and oxidation, very good anti-fouling performance (best used against molten non-ferrous metals, food products, polymers)	700
	**Grey**		
	**Dark grey**		
**TiCN**	**Rose gold**	High hardness, excellent abrasive wear resistance, moderate anti-fouling performance (best used against mechanical galling, metal-on-metal welding, sheet metal debris)	450
	**Copper**		
	**Grey**		
**ZrCN**	**Brown**	High hardness, excellent wear resistance, good corrosion resistance, moderate anti-fouling performance (best used against mechanical galling, metal-on-metal welding, sheet metal debris)	400
	**Grey**		
	**Dark grey**		
	**Blue grey**		
**TiAlN**	**Dark purple**	Excellent hardness, chemical stability, oxidation resistance, and heat resistance, low anti-fouling performance	900
	**Dark grey**		
	**Black**		
**TiAlCrN**	**Matte black**	Excellent toughness and hardness, low friction factor, excellent oxidation resistance, very good anti-fouling performance (best used against molten non-ferrous metals, food residues, polymers)	1000
**Cr**	**Chrome**	Good hardness, excellent toughness and corrosion resistance, low-to-moderate anti-fouling performance (best used against general industrial residue)	400
**DLC**	**Anthracite**	Very low friction, very high hardness, resistant to sliding wear, biocompatible, excellent anti-fouling performance (prevents adhesion of polymers, glues, organic matter, calcium scales)	350
	**Black**		

**Table 3:** Comparative assessment of main color generation strategies, representative coatings and architectures, color-formation mechanisms, angular dependence, key advantages and limitations, typical sputtering approaches and applications.

Color-Generation Strategy	Representative Coatings/Architectures	Color Origin	Color Tunability and Purity	Angular Dependence	Advantages and Limitations	Typical Sputtering Approaches	Typical Applications
Intrinsic-color coatings	TiN, ZrN, CrN, TiCN, ZrCN, TiAlN, TiAlCrN, DLC	Optical response determined by electronic structure, composition, and absorption/reflection behavior of the coating material	Limited color range; color strongly dependent on composition, stoichiometry, and structure	Very low (generally angle-independent)	Excellent durability, wear resistance, scalability, and industrial maturity; limited color diversity	DC/pulsed DC reactive sputtering; alloying and multilayer strategies	Cutting tools, automotive components, consumer goods, architectural coatings, household fixtures
Dielectric interference coatings (DBR and A-DBR)	High/low-refractive-index multilayers (e.g., TiO_2_/SiO_2_, ZrO_2_/SiO_2_)	Constructive and destructive interference from periodic dielectric layers	Very high color tunability and high purity through thickness and refractive-index control	High for DBR (color changes with viewing angle due to optical path variation); low for aperiodic-DBR	Excellent performance; requires precise numerous layers, precise thickness control and long deposition times	Sequential sputtering, reactive sputtering, multilayer deposition	Decorative coatings, optical components, photonic devices
Thin-film absorbers (AM/DAM)	TiAlN/SST, Ge/Au, AlN/Si/Al, SiO_2_/Si/SST	Resonant absorption in an ultrathin lossy dielectric combined with interference from a reflective substrate	High color purity with relatively simple architectures	Low	Short deposition times, reduced material consumption and excellent scalability; protective dielectric layers can improve wear and environmental resistance	Single-target reactive sputtering, multiple-target sputtering, pulsed reactive gas sputtering	Decorative coatings, consumer electronics, architectural surfaces
Metal–insulator–metal (MIM)	Cr/SiO_2_/Cr, Ag/SiO_2_/Ag, Au/SiO_2_/Al, Ni/SiO_2_/Al	Resonant absorption and interference effects in thin-film cavities	High saturation and broad color tuning by controlling dielectric thickness and metal selection	Moderate (design-dependent, increases for dielectric/metal multilayer designs)	Compact architecture and high color saturation; fabrication requires accurate control of nanometric layers and interface quality	Sequential sputtering, multilayer deposition	Decorative electronics, optical filters, advanced functional surfaces
Plasmonic nanoparticle coatings	Au, Ag, Cu, TiN, ZrN, HfN nanoparticles in dielectric matrixes	Localized surface plasmon resonance (LSPR) of metallic nanoparticles	Wide color palette with nanoscale control; color depends strongly on particle size, shape, and distribution	Moderate (affected by nanoparticle morphology and orientation)	Enables multifunctionality (e.g., optical sensing, anti-microbial effects); fabrication complexity and thermal stability remain challenges	Co-sputtering, sequential sputtering, plasma gas condensation, nanoparticle embedding strategies	Smart decorative coatings, photonic devices, security features, watches, and luxury consumer goods
Hybrid photonic–plasmonic coatings	MIM + plasmonic nanoparticles, nanohole arrays, plasmonic nanocomposites	Coupled interference and plasmonic resonances	Highest design flexibility, enabling high color purity, full visible color gamut and reduced angular dependence	Low–to–high (depends on architecture; requires optimization to balance optical stability and functionality)	Promising for next-generation applications; increased complexity in design, deposition, and scale-up; lower industrial maturity	Multilayers, co-sputtering, sequential sputtering, reactive sputtering, template-assisted and template-free sputtering	High-end decorative coatings, photonic devices, anti-counterfeiting, smart surfaces

**Table 4:** Comparative assessment of adaptive high-temperature self-lubricating coating systems, highlighting their lubrication mechanisms, processing routes, operating temperature, service life, fabrication complexity, limitations and representative coating architectures.

Adaptive Mechanism	Coating System	Representative Coating Architectures	Typical Sputtering Method	Effective Temperature	Service Life	Fabrication Complexity	Main Advantages	Main Limitations	Typical Applications
Structural transformation	TMD-based coatings	MoS_2_-Ti [218], MoSe_2_ [241], TiN-WS_2_ [242], WSN [222,243,244], MoSe_x_/WS_x_ multilayer and nanocomposites [245], WS_2_-B [246], MoS_2_/Ta/WB_2_ nanomultilayer [247], MoS_2_ with WB_2_ [248], MoSN [249], Mo-Se-C [250], WS-CF [251], WSC [252], MoSeN [218]	Reactive sputtering, co-sputtering, sequential sputtering	RT–500 °C (up to ~600 °C for optimized architectures)	Moderate	Moderate	Extremely low friction, excellent vacuum performance, rapid adaptive response	Oxidation, humidity sensitivity, relatively low hardness	Aerospace, space mechanisms, precision tribological components
	DLC-based coatings	a-C:H and hydrogen-free DLC [253], Si-W-DLC [254], Si-DLC [255], CrAl-C [256], TiN-DLC [257], B-DLC [258], TiAlN-DLC [259], SiN-Ti-DLC [260], W-DLC [253]	Reactive sputtering, HiPIMS, co-sputtering	RT–400 °C (up to ~500 °C for doped coatings)	Moderate	Moderate	Low friction, high hardness, excellent wear resistance	Graphitization and oxidation at elevated temperatures	Automotive, molds, bearings
	Hybrid carbon/TMD coatings	Si/Ni-DLC and Si/Ta-DLC composite, DLC/MoS_2_ [261], MoS_2_:a-C multilayers [262], DLC/MoS_2_ [263], WS_2_-DLC/DLC multilayer films [261]	Co-sputtering, multilayer sputtering	RT–600 °C	Moderate–high	High	Improved environmental adaptability, combined lubrication mechanisms	Complex architecture and phase optimization	Multienvironment tribological applications
	Oxide-encapsulated lubricating coatings	Al_2_O_3_-MoS_2_ [264], TiO_2_-WS_2_ [265], ZrO_2_-TMD composites [266]	Reactive co-sputtering, sequential sputtering	600–1000 °C	High	Very high	Excellent oxidation resistance and high-temperature stability	Challenging process optimisation	Aerospace, turbines, high-temperature sliding
Metal diffusion	Ag-containing adaptive coatings	MoN-Ag [267], CrAlN-Ag [268], TiAlSiN-Ag [231], NbCN-Ag [269], Mo_2_N-SiN_x_/Ag-SiN_x_ [250], Mo_2_N-Ag/SiN_x_ [212], MoVN-Ag [270], MoNbN-Ag [271], Ag/Mo multilayers [272], CrAlNAgO [273], Ag_2_CrO4 [274], NiCr(MoNb)_x_-Ag [275], TiAgN/W_2_N [276], Ta-Ag [277]	Reactive co-sputtering, HiPIMS, multilayer sputtering	300–900 °C	High	High	Continuous lubricant replenishment, wide operating window	Ag depletion, agglomeration and evaporation	Cutting tools, bearings, aerospace
	Other soft-metal systems	TiN-Cu [278], AlCrN/Cu [279]	Reactive sputtering, hybrid PVD	300–900 °C	Moderate–high	High	Effective high-temperature lubrication	Lower hardness, limited long-term reservoir	High-temperature tribological systems
Tribo-oxidation	Vanadium-containing coatings	VN [280], (V,Mo)N [281], WVN [282], CrVN [283], AlCrSiN/VN [284], TiAlSiCN/VSiN [285], TiAlVN [286], V-containing HEA (e.g., VTiCrMo [287] and TiAlNiVN [288])	Reactive sputtering, multilayer sputtering	500–900 °C	High	High	Formation of lubricious vanadium oxides, excellent high-temperature lubrication	Strong dependence on oxidation kinetics	Dry machining, hot forming
	Tungsten- and molybdenum-containing coatings	W-N [289], CrAlMoN [290], Mo-N [291], CrN-Mo2N-MoSx [292], MoCN [293], Mo-V-N [289]	Reactive sputtering, co-sputtering	500–900 °C	High	High	Stable tribo-oxide formation, good wear resistance	Oxide volatility at very high temperatures	Cutting tools, severe sliding
	Chameleon coatings	TiAlSiN/VSiN [285], TiAlSiN-based adaptive coatings [294], multiphase nitride/oxide architectures [295]	Reactive sputtering, multilayer deposition	RT–900 °C	Very high	Very high	Sequential activation of different lubrication mechanisms	Highly complex design and optimization	Aerospace, gas turbines, extreme environments
Hybrid adaptive systems	Multimechanism adaptive coatings	Ag-containing chameleon coatings [64], oxide/nitride-encapsulated Ag/TMD systems [296,297], TiSiBCN nanocomposites [298], CrAlN + MoWS coatings [299], MoS_2_-CaF_2_-Ag [64]	HiPIMS, reactive co-sputtering, sequential sputtering	RT–1000 °C	Very high	Extremely high	Broadest operating window through sequential activation of multiple lubrication mechanisms	Highest deposition complexity and cost	Next-generation multifunctional coatings for extreme environments

**Table 5:** Comparison of thin-film temperature sensors tested during machining, including cutting conditions, sensor architecture, fabrication route, calibration range, and maximum measured temperature. Cutting conditions are presented as cutting speed Vc in m.min^−1^, feed f in mm.rev^−1^ and depth of cut ap in mm.

Sensor Type and Materials	Architecture	Cutting Conditions (Vc; f; ap)	Calibration Range (°C)	Workpiece Material	Maximum Temperature (°C)	Fabrication Route
TiAlN and ZrAlN NTC thermistor [304]	WC insert; AlN insulation; TiAlN wear protection	80; 0.293; 1.0	400	Ti-6Al-4V titanium alloy	210	DC sputtering; Kapton^®^ shadow masks
TFTC, reported as K-type, Ni/Ni-Cr [306]	Al_2_O_3_ insert; HfO_2_ insulation; TiN wear protection	960; 0.1; 1.0	Not disclosed	A6061 aluminum alloy	320	DC sputtering; helicon sputtering; photolithography
TFTC, Cu/Ni [307]	Al_2_O_3_ insert; Si_3_N_4_ protective/insulating layer	405; 0.25; 2.0	100	A5056 aluminum alloy	500	Ion plating; electro and electroless plating; lapping
C-type TFTC, W-Re [308]	PCBN insert; insulation/protection architecture	90; 0.1; 1.0	300	Ti-6Al-4V titanium alloy	230	RF sputtering; E-beam evaporation; diffusion bonding; photolithography
C-type TFTC, W-Re [309]	PCBN insert; Al_2_O_3_ insulation layer	800; 0.1; -	125	A6061 aluminum alloy	120	DC sputtering; E-beam evaporation; diffusion bonding; photolithography
N-type TFTC, reported as NiCr/NiSi [310]	PCBN insert; no additional layers	45; 0.16; 0.5	1000	A3 tool steel	60	DC sputtering; metallic shadow masks
TFTC, Ni/Ni-Cr [311]	Si_3_N_4_ insert	580; 0.2; 0.2	Not disclosed	Ti-10C-2Fe-3Al titanium alloy	285	Cathodic arc; Metallic shadow masks
TFTC, K-type chromel–alumel [312]	WC insert; Si_3_N_4_ protective/insulating layer	80; 0.2; 0.4	300	Ti-6Al-4V titanium alloy	450	Plasma-enhanced CVD; DC sputtering; photolithography; laser machining
TFTC, K-type chromel–alumel [313]	WC insert; Si_3_N_4_ protective/insulating layer	50; 0.1; 0.5	300	Ti-6Al-4V titanium alloy	120	Plasma-enhanced CVD; DC sputtering; photolithograph; laser machining
TFTC, K-type chromel–alumel [314]	WC insert; Al_2_O_3_ insulation layer	80; 0.2; -	350	Ti-6Al-4V titanium alloy	750	CVD; DC sputtering; photolithography
TFTC, reported as T-type, NiCr and NiSi [316]	WC insert; Si_3_N_4_ protective/insulating layer	60; 0.15; 0.2	300	Ti-6Al-4V titanium alloy	160	Plasma-enhanced CVD; DC sputtering; metallic shadow masks
TFTC, K-type chromel–alumel [317]	WC insert; Al_2_O_3_ insulation; AlTiN wear protection	200; 0.05; 2	100	12L14 steel	120	DC sputtering; RF sputtering; metallic shadow masks
TFTC, K-type chromel–alumel [319]	WC insert; Al_2_O_3_ insulation; AlTiN wear protection	120; 0.075; 2	500	4140 steel	550	DC sputtering; RF sputtering; metallic shadow masks
TFTC, K-type chromel–alumel [320]	WC insert; Al_2_O_3_ insulation; AlTiN wear protection	200; 0.05; 2	500	4140 steel	750	DC sputtering; RF sputtering; metallic shadow masks
Thermoresistive PTC-Cr [324]	WC insert; Al_2_O_3_ protective/insulating layer	100; 0.15; 0.3	200	4140 steel	225	DC sputtering; RF sputtering; photolithography
Thermoresistive PTC-Cr [325]	WC insert; Al_2_O_3_ protective/insulating layer	100; 0.2; 0.6	200	4140 steel	190	Plasma-enhanced CVD; DC sputtering; photolithography
Thermoresistive PTC-Cr [326]	WC insert; Al_2_O_3_ protective/insulating layer	30; -; -	400	4140 steel	260	Plasma-enhanced CVD; DC sputtering; photolithography

## Data Availability

No new data were created or analyzed in this study. Data sharing is not applicable to this article.
